# Engineered nanodrug targeting oxidative stress for treatment of acute kidney injury

**DOI:** 10.1002/EXP.20220148

**Published:** 2023-07-20

**Authors:** Liwen Li, Yining Shen, Zhongmin Tang, Yuwen Yang, Zi Fu, Dalong Ni, Xiaojun Cai

**Affiliations:** ^1^ Department of Ultrasound in Medicine Shanghai Jiao Tong University School of Medicine Affiliated Sixth People's Hospital Shanghai People's Republic of China; ^2^ Departments of Radiology and Medical Physics University of Wisconsin‐Madison Wisconsin USA; ^3^ Department of Orthopaedics Shanghai Key Laboratory for Prevention and Treatment of Bone and Joint Diseases Shanghai Institute of Traumatology and Orthopaedics Ruijin Hospital Shanghai Jiao Tong University School of Medicine Shanghai People's Republic of China

**Keywords:** acute kidney injury, nanomaterials, oxidative stress, reactive oxygen species, renal targeting

## Abstract

Acute kidney injury (AKI) is a clinical syndrome characterized by a rapid decline in renal function, and is associated with a high risk of death. Many pathological changes happen in the process of AKI, including crucial alterations to oxidative stress levels. Numerous efforts have thus been made to develop effective medicines to scavenge excess reactive oxygen species (ROS). However, researchers have encountered several significant challenges, including unspecific biodistribution, high biotoxicity, and in vivo instability. To address these problems, engineered nanoparticles have been developed to target oxidative stress and treat AKI. This review thoroughly discusses the methods that empower nanodrugs to specifically target the glomerular filtration barrier and presents the latest achievements in engineering novel ROS‐scavenging nanodrugs in clustered sections. The analysis of each study's breakthroughs and imperfections visualizes the progress made in developing effective nanodrugs with specific biodistribution and oxidative stress‐targeting capabilities. This review fills the blank of a comprehensive outline over current progress in applying nanotechnology to treat AKI, providing potential insights for further research.

## Graphical abstract

Engineered nanoparticles targeting oxidative stress have shown promising perspectives in treating acute kidney injury (AKI). By examining the challenges, advancements, and limitations of different studies in this field, this review fills a significant knowledge gap. In addition, it offers valuable insights for future research on engineering nanoparticles to address AKI.

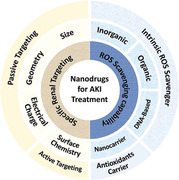

## 1 INTRODUCTION

Although once seen as a specific disease, acute kidney injury (AKI) is now recognized as a syndrome.^[^ ^1^ ^]^ Its occurrence rate is over 50% in intensive care units and as high as 22.7% among hospitalized patients.^[^ ^2^ ^]^ AKI is usually a silent syndrome since it alone does not cause pain or show any specific symptoms, thus complicating early diagnosis. The primary pathological changes are an increase in serum creatinine levels and a decrease in urine output. Based on the creatinine levels and urine output characteristics, the Kidney Disease Improving Global Guidelines proposed a more precise definition of AKI in 2012.^[^ ^2^, ^3^ ^]^ A further significant challenge in diagnosing AKI is that it often coexists with other severe syndromes, such as heart failure, liver failure, and sepsis, which then increases related rates of morbidity and mortality. It is thus easy to overlook the clinical significance of AKI.^[^ ^1^ ^]^ Currently, the prime principle in the clinical treatment of AKI is prevention. The first method is to eliminate its cause or trigger, and the second is to avoid further insult.^[^ ^4^ ^]^ Beyond this, the additional focus of medical treatment is mainly supportive, including nutritional support, anaemia correction, metabolic acidosis monitoring, and even renal replacement therapy and extracorporeal support for the management of severe AKI.^[^ ^5^ ^]^ Unfortunately, there is no well‐implemented treatment that can reverse or stop the process of AKI development. Thus, the central focus is on the prevention of further renal damage. When further insult is inevitable (e.g., chemotherapy), a level‐one treatment for preventing AKI development is desirable; there is thus currently a need for a novel and effective treatment for AKI.

Considerable research has proven that the occurrence of excess reactive oxygen species (ROS) is closely related to renal damage and this can be an activator of AKI.^[^ ^6^ ^]^ Studies have also shown that ROS are significant inducers of drug‐induced renal damage, ischemia‐reperfusion injury, diabetic nephropathy, and other AKI pathologies. The primary sources of ROS in the kidney are nicotinamide adenine dinucleotide phosphate (NADPH) oxidase and mitochondria, which normally produce benign levels of ROS, but these increase dramatically when pathophysiologically stimulated.^[^ ^7^ ^]^ Physiologically, average levels of ROS, such as peroxides, superoxides, singlet oxygens, or hydroxyl radicals, are crucial for cell metabolism, including antioxidant system signalling and the evasion of hypoxia, and ROS are not harmful during these processes. Stimulations, such as sepsis, major surgery, hypovolemia, and renal inflammatory processes, however, can result in excess ROS generation and thus oxidative stress, as they can affect intracellular regulatory systems.^[^ ^8^ ^]^ Additionally, oxidative stress can lead to decreased levels of antioxidants, such as superoxide dismutase (SOD), which further increases the level of stress. A reduction in ATP synthesis can be observed during this process, and if it is severe, it can lead to various clinical symptoms.^[^ ^9^ ^]^ Even if renal antioxidant systems, including the SOD, catalase (CAT), peroxidase (POD), and glutathione peroxidase (GPx) systems, can effectively reduce the ROS level in the kidney, arenal injury may occur when oxidant stress exceeds the ability of the antioxidant systems to counterbalance ROS levels.^[^ ^10^ ^]^

Previous studies have shown that antioxidants can reduce ROS production and protect the kidney to a certain extent, which is promising for the development of novel therapies for AKI.^[^ ^11^ ^]^ Unfortunately, clinical antioxidant treatments for AKI are still limited, and there are many potential reasons for this. One reason is that antioxidants may have low bioavailability, and the time and duration of antioxidant usage may not be optimal. While another is that the antioxidants may have poor target specificity.^[^ ^12^ ^]^ ROS are also crucial for normal cell function and metabolism, and an overall elimination would certainly affect internal signalling and lead to cellular injury.^[^ ^10^ ^]^ In addition, various ROS sources and internal dynamic connections can obstruct effective antioxidant therapy development.^[^ ^13^ ^]^ Consequently, the development of an antioxidant that explicitly targets mitochondrion dysfunction or related cells and has good bioactivity could lead to the development of a novel clinical treatment for AKI.^[^ ^14^ ^]^

**FIGURE 1:**
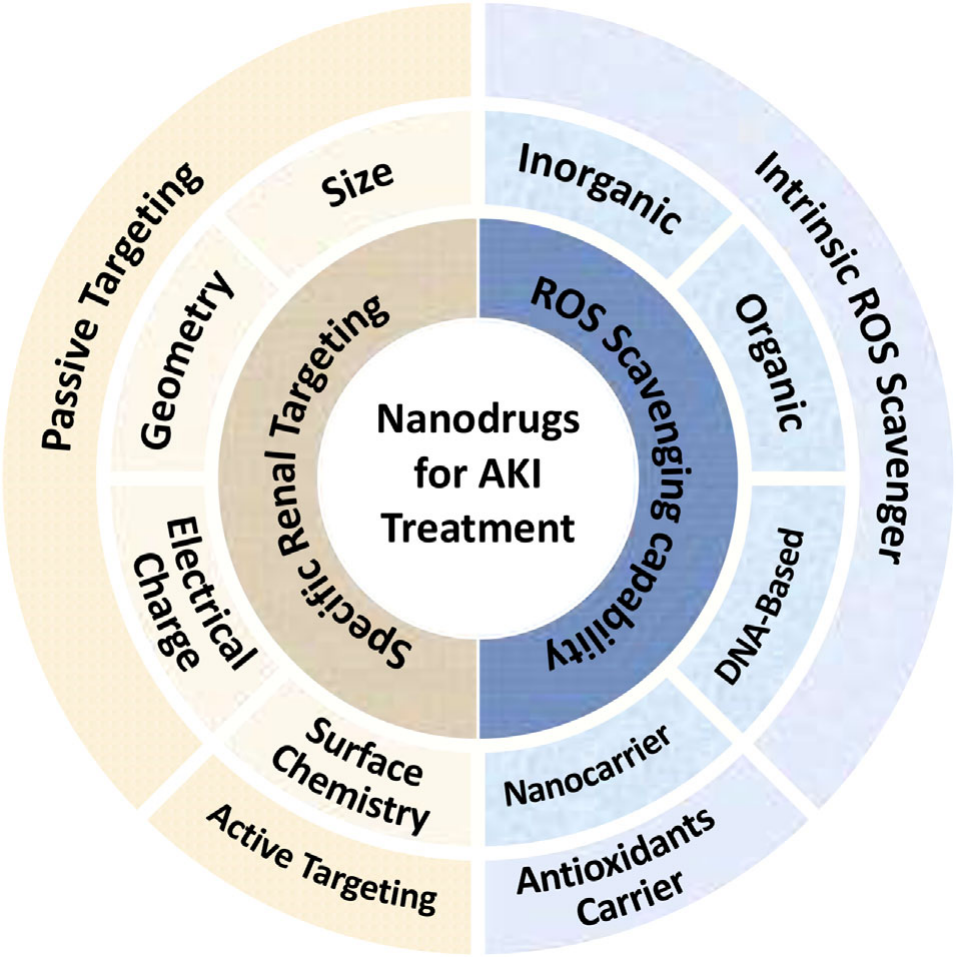
Schematic diagram showing the current research fields involved in developing nanodrugs targeting oxidative stress for AKI.

Nanotechnologies have experienced considerable prosperity and development in recent decades,^[^ ^15^ ^]^ including a broad range of engineered nanoparticles for treating AKI (Figure 1).^[^ ^16^ ^]^ The powerful nanotechnology‐based tools enable nanoparticles to be engineered with desired physicochemical properties by adjusting their size, shape, and surface.^[^ ^17^ ^]^ To be clinically practical, nanodrugs must have the ability to specifically target, be medically effective or capable of loading efficacious medicine, be minimally bio‐toxic, and be easily mass‐produced. While many engineered nanoparticles have been shown to be promising, comprehensively grasping the current progress and issues to be addressed can be difficult. Several reviews have focused on nanomedicines of specific components or functionalities; however, a comprehensive review of current progress in applying nanotechnology to treat AKI remains unfulfilled. This review has aimed to summarize the current state of different research fields working to engineer nanodrugs to target oxidative stress and treat AKI (Table 1), thus identifying currently unsolved problems and the required directions for future research. First, we introduce the AKI pathology and the engineered nanodrugs passively or actively targeting the glomerular filtration barrier (GFB) in AKI treatments. Then, engineered nanodrugs with ROS scavenging properties for treating AKI are discussed in detail. Finally, several aspects necessary for the clinical application of engineered nanodrugs for treating AKI are discussed. Readers can check these two comprehensive reviews in terms of experimental models and the imaging of AKI for translational research.^[^ ^18^ ^]^

## 2 PATHOLOGY OF ISCHEMIA/REPERFUSION‐INDUCED RENAL‐AETIOLOGY AKI

AKI can be categorized into three main aetiologies: prerenal, intrinsic, and postrenal.^[^ ^19^ ^]^ Recent studies have mainly focused on the renal‐aetiology of AKI, and consequently, the pathology of the ischemia‐induced renal‐aetiology of AKI will be the focus of this section. However, it should be noted that the mechanisms of the other types of AKI are readily accessible in the scientific literature.^[^ ^20^ ^]^ The combinative effects of renal tubular epithelial cells, together with innate and adaptive immune responses of immune cells, contribute to the pathology of intrinsic AKI. Throughout the pathological process, inflammatory‐associated molecular patterns, hypoxia‐inducible factors, complement systems, oxidative stress, and cell death all significantly impact renal functionality. Researchers have thus developed various medicines targeting inflammatory, apoptosis, and oxidative‐related stresses. This section focuses on the oxidative stress pathway, a crucial target in nanodrug development.^[^ ^21^ ^]^

In physiological states, oxidants and antioxidants are in a dynamic equilibrium, maintaining the standard functionality of renal cells. However, due to external factors, such as ischemia/reperfusion or the presence of renal toxic chemicals, the delicate balance is broken, resulting in excessive ROS formation, which leads to oxidative stress in the extracellular environment, thus stimulating the recruitment of numerous inflammatory cells and the production of inflammatory cytokines, such as transforming growth factor‐beta (TGF‐β), vascular endothelial growth factor (VEGF), plasminogen activator inhibitor‐1 (PAI‐1), and monocyte chemotactic protein‐1 (MCP‐1).^[^ ^22^ ^]^ As inflammatory states endure, mitochondrial dysfunction, lipid peroxidation, DNA damage, and protein nitration can occur, catalysing further cellular injury. Moreover, the mitochondria can produce excessive ROS in stress states, thus magnifying oxidative stress after disintegration.^[^ ^23^ ^]^

Additionally, throughout ischemia, the proportion of the blood flow towards the inner and outer medulla is reduced much more significantly than the total renal blood flow (RBF) reduction, suggesting inner dysfunction with AKI.^[^ ^24^ ^]^ Endothelial injury occurs in ischemia, and during this process, the levels of several vasoconstrictive hormones and cytokines (e.g., angiotensin II, thromboxane A2, and leukotrienes C4 and D4) are increased.^[^ ^25^ ^]^ These activated leukocytes then migrate through the gap between endothelial cells and the extracellular matrix, causing the matrix to expand.^[^ ^26^ ^]^ The combined effect results in small renal arterioles vasoconstriction, thus further compromising local blood flow and microcirculation, which forms a vicious cycle and reduces RBF. In ischemia/reperfusion‐induced AKI, impaired GFB could allow larger particles to pass through, thus allowing more sizeable nanodrugs to accumulate specifically in the kidney. Additionally, macrophage proportion and certain surface receptor levels are elevated in the pathology state, which enables active renal targeting strategy in nanodrug development.

## 3 ENGINEERED NANODRUGS TARGETING THE GFB IN AKI TREATMENTS

**FIGURE 2:**
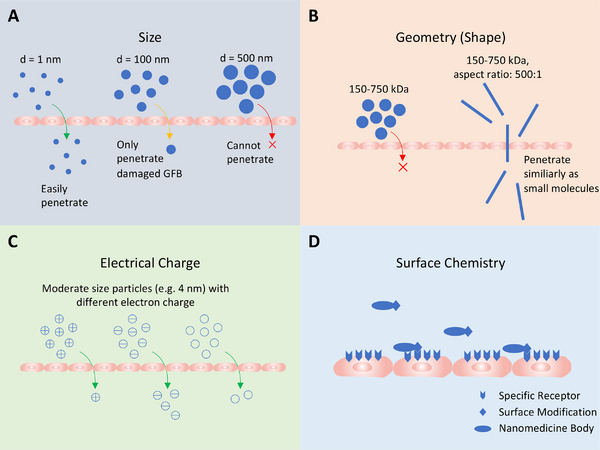
Targeting factors for nanomedicines around the glomerular filtration barrier (GFB) in AKI treatments: (A) size: size is the most crucial factor in GFB targeting, as larger nanomedicines are less likely to penetrate the GFB; (B) geometry: in vivo convective forces are also likely to affect nanomedicines, thus making geometry properties crucial; (C) electrical charge: large positively charged molecules receive more hindrance when penetrating the GFB than neutral particles, and negatively charged molecules penetrate most easily; (D) surface chemistry: in AKI, some cell surface receptors, or other proteins may be overexpressed, and could thus be utilized as specific targets in the development of nanomedicines.

Traditional clinical treatments for AKI are mainly supportive, and the small molecule compounds utilized have non‐specific biodistributions, which can lead to significant adverse effects. Previous studies have confirmed that targeting the GFB is a promising way to improve the biosafety and efficacy of these drugs.^[^ ^16b^ ^]^ Nanoparticles provide more engineering and modification possibilities, making GFB‐targeting practical. Successfully targeting the kidney or renal cells is crucial for developing nanomedicines suitable for clinical use. Current targeting methods are clustered into two major groups: passive targeting (size, shape, and electrical charge) and active targeting (surface chemistry) (Figure 2).

### 3.1 Passive targeting: Size, shape, and charge

Normal kidneys, which are approximately the size of a fist, produce an enormous 180 L of glomerular filtrate per day. The filtrate contains primarily inorganic ions and small organic molecules, while large proteins, platelets, and cells cannot penetrate the GFB. The GFB consists of three layers, including the endothelial cells of the capillaries, which are responsible for maintaining the cells and platelets, capillary basement membrane, and epithelial cells (podocytes). GFB filters molecules selectively according to their size and electrical charge.^[^ ^27^ ^]^ For molecules <7000 Da (estimated 2.53 nm in diameter)^[^ ^28^ ^]^, including most small ions, urea, glucose, amino acids, and other small molecules, the GFB provides no hindrance. In contrast, the GFB excludes most solutes >70,000 Da (estimated 5.44 nm in diameter).^[^ ^28^ ^]^ Molecules with 7000–70,000 Da are filtered progressively less as the molecule size increases. Electrical charge is the second dominating factor that determines the amount of filtration. For any given molecule size, negatively charged macromolecules almost always receive more hindrance than neutral particles, while increased amounts of positively charged molecules penetrate the GFB.^[^ ^27^ ^]^ With a properly designed shape, however, particles significantly larger than 70,000 Da may still penetrate the GFB and thus reach the endothelial cells.

#### 3.1.1 Size

Nanodrugs less than 2–5 nm can penetrate GFB freely and reach endothelial cells, leading to renal accumulation; this is the foundation for practical medical effects. Furthermore, tiny particles can rapidly degrade, which obviates the severe adverse effects caused by long‐term accumulation. Most researchers use transmission electron microscopy (TEM) to measure the size of engineered nanodrugs and apply in vivo positron emission tomography (PET) or fluorescence imaging to demonstrate the biodistribution inside experimental animals.

Molybdenum‐based polyoxometalate nanoclusters have an average diameter of 1 nm and a hydrated size of <10 nm, which means they can pass through the kidneys, resulting in very high and specific levels of renal uptake.^[^ ^29^ ^]^ Additionally, Zhang et al. engineered biodegradable self‐assembled ultrasmall nanodots that consist of iron ions, gallic acid, and polyvinylpyrrolidone (PVP). These particles have a diameter of 1−2 nm and a thickness of approximately 3 nm. In vivo fluorescence images show that they accumulated in the renal area and were degraded after approximately 24 h.^[^ ^30^ ^]^ These characteristics increase efficiency and reduce adverse effects, which is necessary for clinical usage. However, not all tiny nanoparticles have high renal accumulation. For example, ultrasmall Mn^2+^‐chelated melanin nanoparticles, with a similar hydrodynamic size of approximately 4.5 nm, were found to have dramatically different levels of biodistribution. These particles had exceptionally high accumulation in the liver and heart area, and while renal uptake was observed, it was overwhelmed by the accumulation in other organs.^[^ ^31^ ^]^ The exact reason why these particles with similar sizes have very different biodistributions remains uncertain. Possible explanations include differences in their electrical charges and surface chemistry.

**FIGURE 3:**
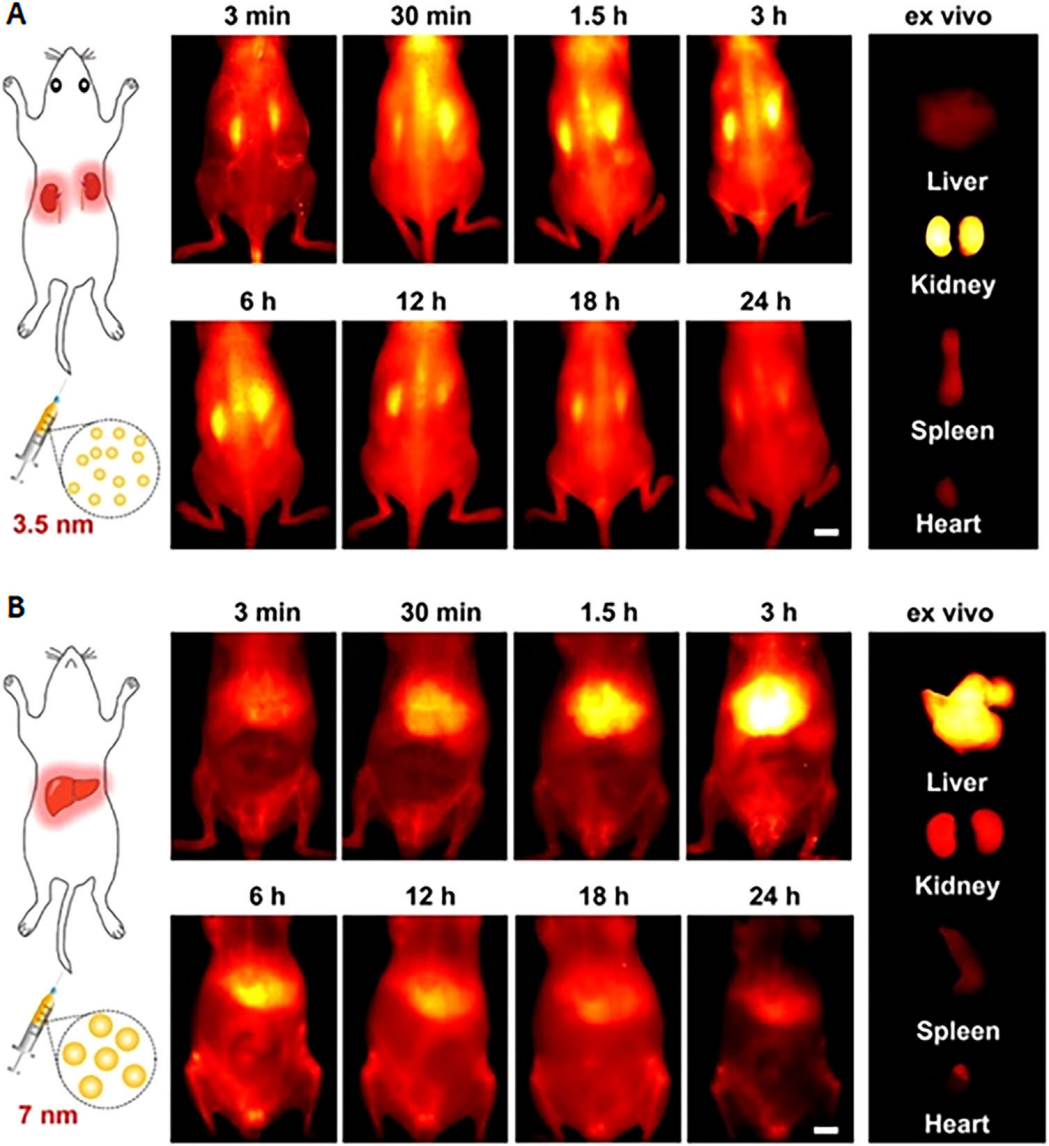
In vivo and ex vivo fluorescence imaging demonstrates that size is a significant factor influencing the biodistribution of nanoparticles: (A) nanodots of 3.5 nm display specific renal accumulation; (B) similar nanodots of 7 nm exhibit significant liver uptake and lesser renal amassing. Reproduced with permission.^[^ ^32^ ^]^ Copyright 2021, American Chemical Society.

In many cases, nanoparticles can accumulate in both the liver and kidney. A research team constructed a nanoprobe based on black phosphorus quantum dots, which exhibit different biodistributions depending on size. In vivo fluorescence imaging displayed very different biodistribution of the nanoparticles with similar components but different sizes: nanoprobes of approximately 3.5 nm show specific renal uptake, while particles of approximately 7 nm display significant liver accumulation (Figure 3). This controlled experiment corroborates the idea that size is the most determining factor in GFB targeting.^[^ ^32^ ^]^

**FIGURE 4:**
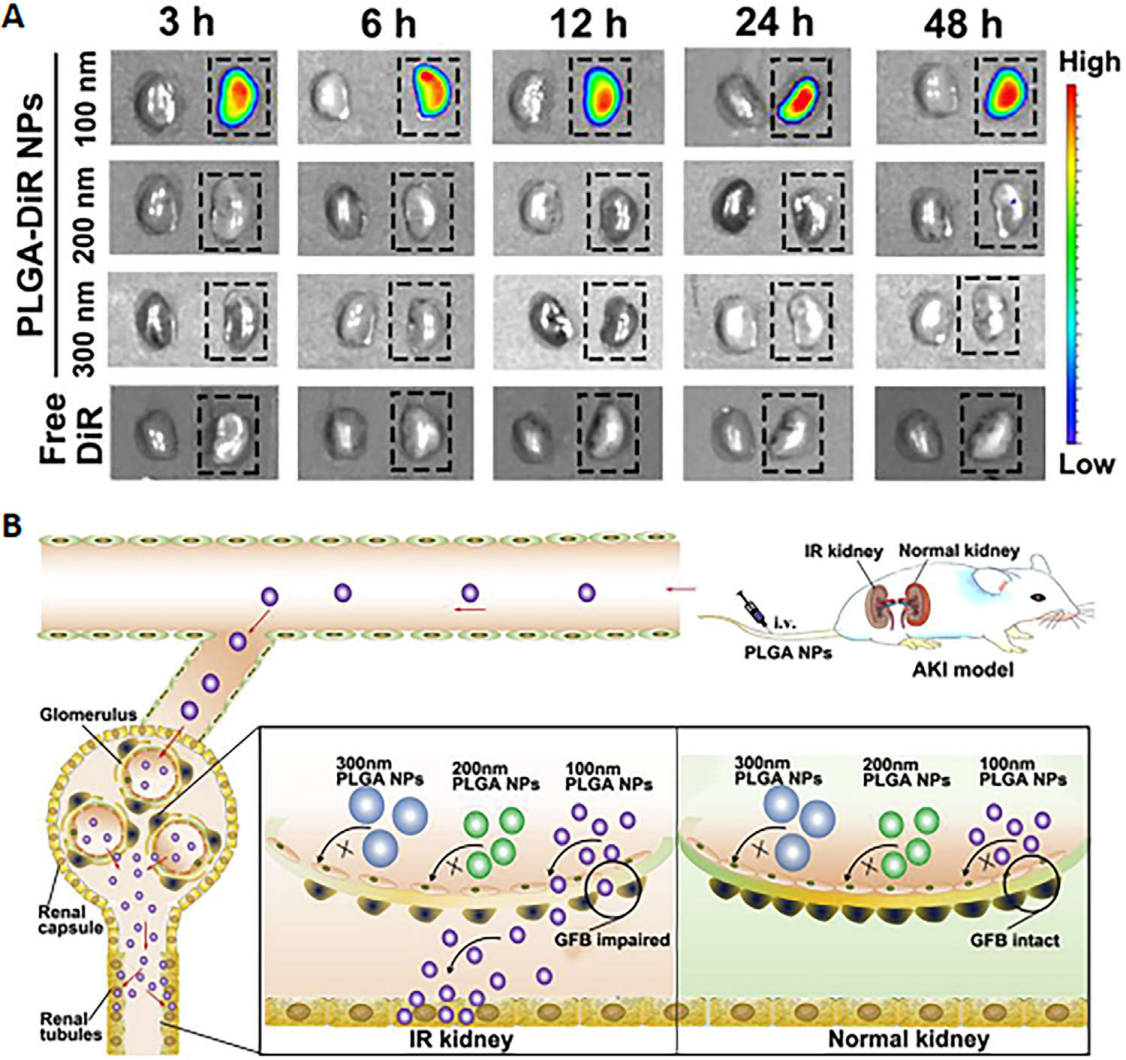
Comparative experiments over nanomedicines of different sizes (100 nm, 200 nm, and 300 nm): (A) NIR fluorescence images of injured kidneys (dotted box) and contralateral normal kidneys at different times (3, 6, 12, 24, 48 h) after injection of different sized nanoparticles (100 nm, 200 nm, 300 nm, and free DiR as control). The figure shows no renal uptake in normal kidneys, while 100 nm nanodrugs could penetrate GFB and accumulate in injured kidneys. (B) Schematic diagram to explain why 100 nm particles could penetrate GFB despite that typical GFB only allows particles less than 10 nm. The research constructed poly (lactic‐co‐glycolic acid) nanocarriers loaded with Oltipraz to carry the antioxidative molecules to the specific site. Reproduced with permission.^[^ ^33^ ^]^ Copyright 2019, Elsevier.

Small particles are more likely to aggregate in the kidney, but it is also possible for large nanodrugs to penetrate the GFB. Another study has suggested that AKI may cause changes to the structure and permeability of the GFB, thus providing opportunities for larger nanomedicines to accumulate in the renal area.^[^ ^17^ ^]^ In this study, particles of 100, 200, and 300 nm were constructed. In vitro fluorescence images show significant renal uptake of the 100 nm particles in the ischemia/reperfusion kidneys, while there was no observed accumulation of the 200 or 300 nm particles. (Figure 4A)^[^ ^33^ ^]^ A possible explanation for this phenomenon is that an intact GFB can only filter particles <10 nm, while a GFB impaired by AKI would allow much larger particles to be filtered (Figure 4B). This exciting discovery means that complex nanodrugs, such as nanocarriers, could be utilized to carry antioxidants towards impaired renal cells. However, when particles become larger, they also become harder to degrade, which makes biosafety another primary concern, and consequently, more research is required to address this problem.

#### 3.1.2 Geometry (Shape)

In vivo convective forces have a far more significant impact on nanoparticles than Brownian motion^[^ ^34^ ^]^, thus making the shape of nanomedicines crucial to their biodistribution and clinical effects.^[^ ^27^, ^35^ ^]^ While technically, all objects in our world are three‐dimensional, we can borrow the concept of different dimensions to illustrate different shapes of engineered nanodrugs as follows: 0D, 1D, and 2D.

Nanoparticles that are small spheres can be regarded as 0D, and this includes all the different‐sized particles previously mentioned. Since the different convective forces from the different aspects are identical within this dimension, the geometric engineering possibilities are not very exciting. Apart from the exceptional circumstances mentioned previously, smaller nanodrugs have a higher chance of penetrating the GFB and accumulating in the renal area.

As the dimensions grow beyond zero, things become more interesting. 1D space is a line. Thus, a stick‐like nanoparticle can be defined as a 1D nanodrug. Relatively long nanoprobes with small diameters can provide a more complex structure and still penetrate GFB freely. Ruggiero et al. have found that nanoparticles that are 100−1000 nm in length with a diameter of 0.8−1.2 nm can easily penetrate the GFB. The researchers constructed single‐walled carbon nanotubes with ligands for in vivo near infrared (NIR) imaging and discovered that molecules with aspect ratios as high as 500:1 could surprisingly penetrate the GFB similar to the smaller molecules. The team found that nanotubes with <500:1 were preferred and that forces tend to orientate them to the GFB (caused by solid flow) and this is capable of overwhelming the rotational diffusion (caused by dash curves and Brownian motion), thus making the nanotubes highly oriented towards and quickly cleared by the GFB.^[^ ^36^ ^]^ Other researchers established an ammonium‐functionalized carbon nanotube^[^ ^37^ ^]^ as a transportation media for small interfering RNA (siRNA). Positron emission tomography‐computed tomography results showed that the accumulation of siRNA transported via carbon nanotubes was ten times higher in the renal area than that of the siRNA alone, thus proving the renal targeting effects of the carbon nanotube.^[^ ^38^ ^]^

**FIGURE 5:**
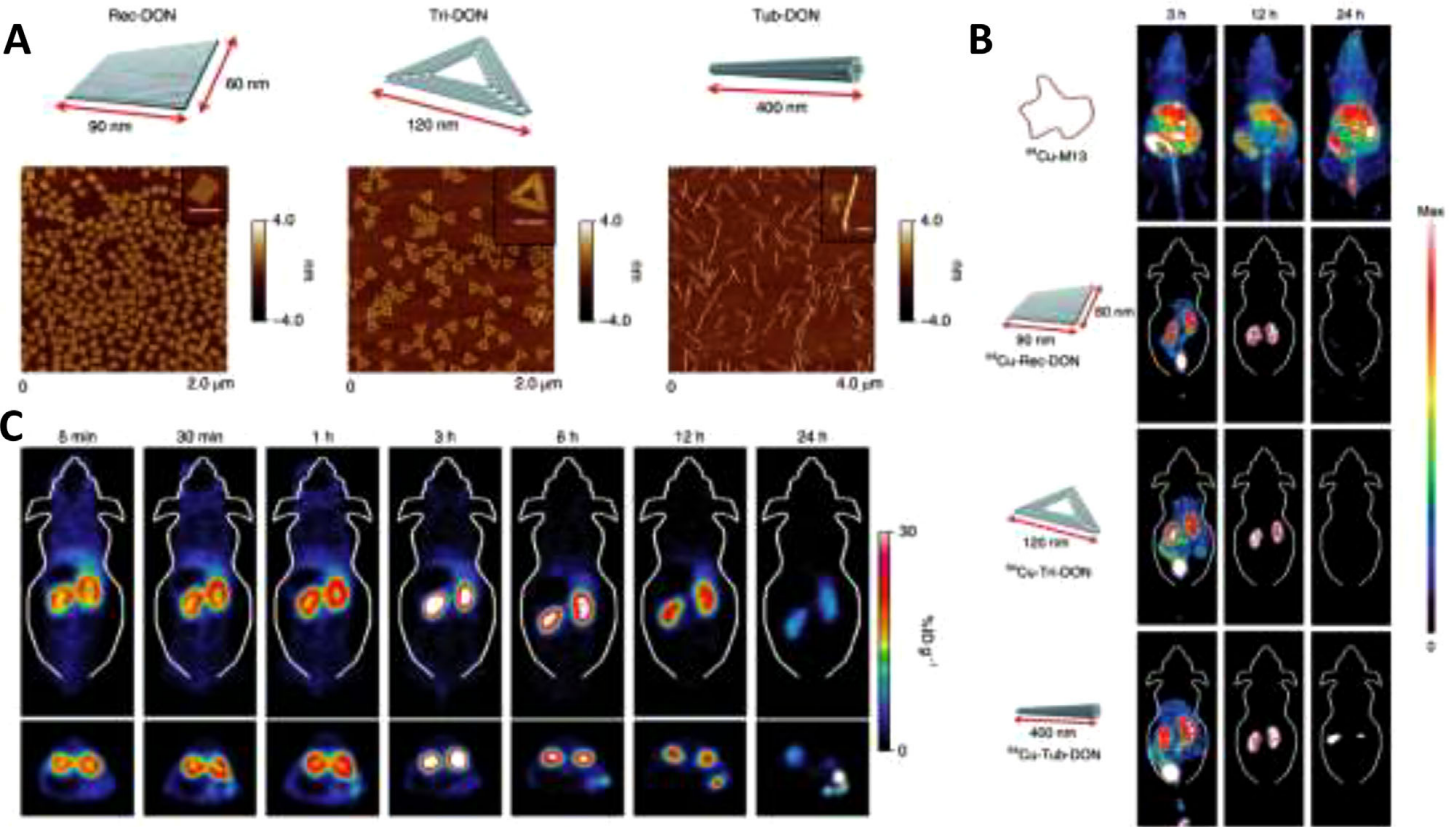
DNA origami nanostructures of different shapes displaying various renal targeting capabilities: (A) Schematic diagram of three DNA origami nanostructures and corresponding atomic force microscope images (scale bars: 100 nm); (B) In vivo comparative PET images, taken at 3, 12, and 24 h post‐intravenous injection, to analyse the biodistribution of M13 and three different nanostructures. The rectangular structure exhibits the most specific renal accumulation; (C) PET image showing biodistribution in the AKI mouse model. The experiments confirmed that kidney injury does not affect specific biodistribution. Reproduced with permission.^[^ ^40^ ^]^ Copyright 2018, Springer Nature.

Two‐dimensional space is a plane. Thus, a sheet‐like nanostructure can be defined as a 2D nanodrug. Thin nanosheets have even more complex structures and can still target GFB specifically. Structural DNA nanotechnologies have been rapidly developing over previous decades, and numerous researchers have developed a variety of predictable, programmable DNA nanomedicines with two and three dimensions.^[^ ^39^ ^]^ Jiang and his team designed rectangular, triangular, and tubular DNA origami nanostructures (DONs) and applied PET imaging to evaluate the in vivo accumulation of the nanodrugs in an AKI mouse model (Figure 5A). To assess the stability of these nanostructures, the team examined 3‐ and 12‐h post‐injection in the urine using fluorescence imaging, and the results showed that some of the structures remained intact after the excretion process. Additional in vitro fluorescence resonance energy transfer assays showed that the efficiencies of the nanostructures decreased to approximately 50% after 12 h of 80% foetal bovine serum (FBS) and 50% mouse urine incubation. Thus, the two results indicate that the well‐folded DON structure probably remains intact before reaching the targeted kidney region.^[^ ^40^ ^]^ Compared with M13 (a circular single‐stranded DNA used as the scaffold for annealing), these DONs display higher levels of kidney accumulation and lower levels of liver uptake in healthy and AKI mice (Figure 5B,C). These results prove that specific renal uptake is mainly attributed to well‐designed 2D structures. Among the three shapes mentioned previously (triangular, rectangular, and tubular), the rectangular DONs have superior in vivo accumulation in AKI mice.^[^ ^40^ ^]^ Thus, carefully selecting a 2D shape for nanodrugs could improve the targeting effect.

#### 3.1.3 Electrical charge

**FIGURE 6:**
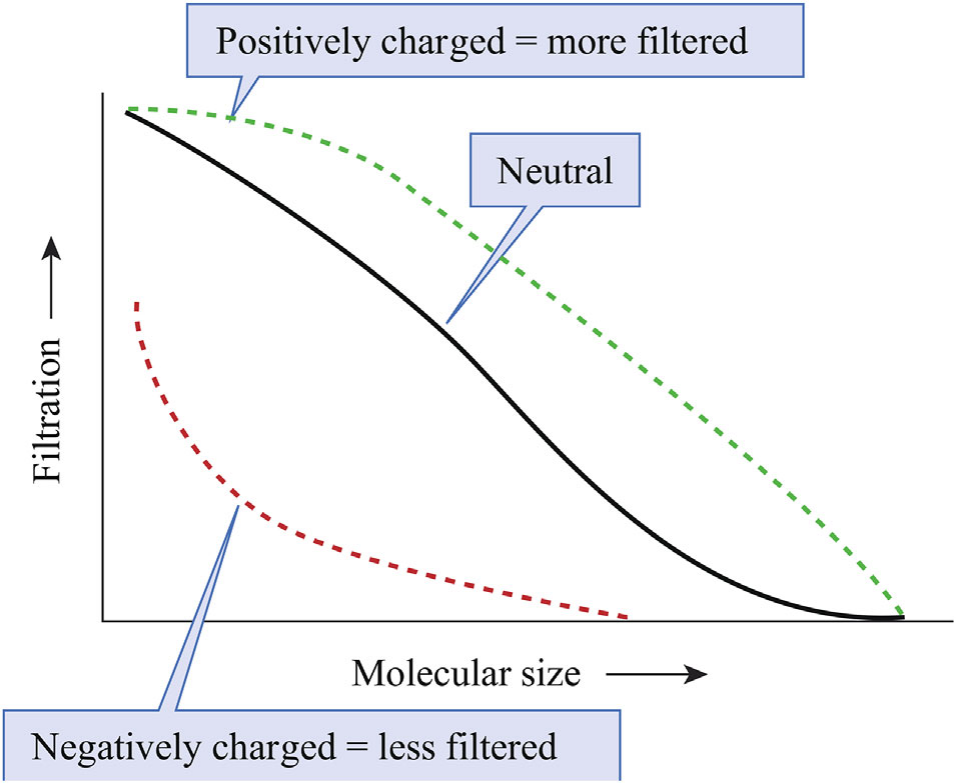
Given the molecule size, negatively charged nanoparticles are more restricted, while positively charged ones are more filtered. Reproduced with permission.^[^ ^44^ ^]^ Copyright 2019, McGraw Hill Education.

For particles larger than the size of albumin, the electrical charge is another dominating factor in glomerular filtration. Positively charged particles are filtered more than negatively charged ones.^[^ ^27^ ^]^ A nanoparticle's size and surface charge significantly impact its GFB filtration rate (Figure 6). However, despite it being more challenging to pass the GFB, most nanomedicines are still designed to be negatively charged because most proteins in the serum are negatively charged. Thus, a negative surface charge can prevent protein corona formation ^[^ ^41^ ^]^ and reduce the interconnection with phagocytes.^[^ ^42^ ^]^ Being negatively charged can also decrease reticuloendothelial system uptake and help to avoid considerable accumulation in the liver and spleen.^[^ ^43^ ^]^ Although negatively charged nanoparticles will receive more hindrance while penetrating the GFB, this factor also extends the staying time inside the renal area, consequently increasing the speed and extent of accumulation in the area. The enhanced amassing is also a potential reason for the previously mentioned two‐dimensional DNA nanostructures to show prolonged (12 h after injection) specific accumulation inside the kidney.^[^ ^40^ ^]^ Rosenkrans et al. developed radiolabelled, highly negatively charged selenium‐doped carbon quantum dots (SeCQDs) with a diameter of 40 nm and a height of 2 nm for AKI treatment. The PET images show that the nanodots can survive inside the kidney for over 72 h, which could be attributed to the highly negative electrical charge.^[^ ^43^ ^]^ Although this attribute can be used to develop long‐term effective medicines, we must emphasize that prolonged accumulation indicates slow degradation, which can lead to severe adverse effects. This characteristic provides researchers with another aspect to consider when engineering novel nanodrugs.

### 3.2 Active targeting: Surface chemistry

**FIGURE 7:**
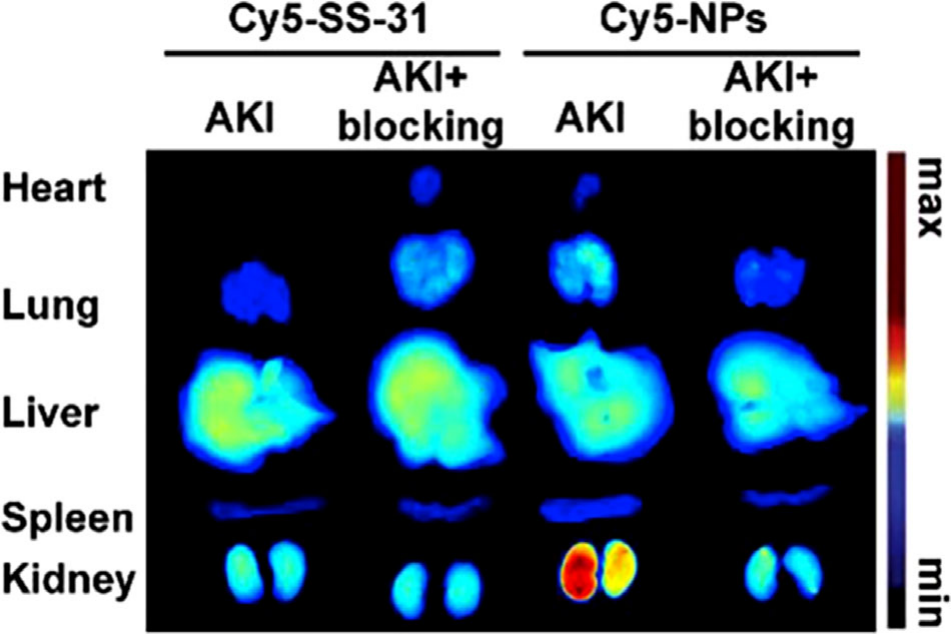
Fluorescence image showing the biodistribution of the nanopolyplexes targeting the CD44 receptor, which is overexpressed in AKI. Cy5‐SS‐31 was used to form Cy5‐nanopolyplexes (Cy5‐NPs). To confirm that the specific accumulation is related to CD44 receptors, researchers pretreated AKI mice with hyaluronic acid to block their CD44 receptors and performed a comparative experiment. In this image, Cy5‐NPs show significant renal uptake in the AKI model, which vanished when the CD44 receptors were blocked. Reproduced with permission.^[^ ^49^ ^]^ Copyright 2019 Elsevier.

Passive targeting engineering strategy takes advantage of the intrinsic properties of the GFB and nanoparticles, while active targeting, though rugged, provides us with far more possibilities for targeting the renal area. Through surface modifications, we can modify the properties of nanomedicines and enhance their abilities to target specific organs, such as the kidneys. Developing proper surface chemistry can be extremely complicated since the properties encountered by in vivo cells (called “biological identity”) can differ significantly from the properties experienced when synthesized in the laboratory (called “synthesis identity”).^[^ ^45^ ^]^ Despite this, many researchers have developed specifically targeting nanomedicines for various diseases.^[^ ^46^ ^]^ Previous studies discovered that with kidney disease, there is an overexpression of CD44 receptors ^[^ ^47^ ^]^, and SS‐31 displays practical ROS scavenging abilities.^[^ ^48^ ^]^ Thus, it was reported that a novel nanoparticle coated with CD44 receptor‐targeting ligands could deliver SS‐31 for treating AKI.^[^ ^49^ ^]^ In vivo fluorescence images demonstrate a significant increase in renal uptake compared to the raw SS‐31. Additionally, the team performed a blocking experiment to confirm that the accumulation is related to the overexpression of CD44 receptors (Figure 7). The nanoparticle also has a pH‐responsive ability, which would rapidly release the carried SS‐31 in acidic conditions.

In AKI, the kidney exhibits an early inflammatory response, with increased M1 macrophage level and partial differentiation towards M2 macrophages. Overexpression of CD163 was observed in M2 macrophages. Thus, Alfonso Rubio‐Navarro et al. developed gold‐coated iron oxide nanoparticles vectorized with an anti‐CD163 antibody. The magnetic resonance imaging (MRI), electron microscopy, and immunological study results jointly confirmed the specific renal targeting of the nanoparticles.^[^ ^50^ ^]^ With ingeniously designed surface modification, the specific targeting and proper drug release attributes make it possible to engineer ideal nanomedicine and nanocarriers.

## 4 ENGINEERED NANODRUG WITH ROS‐SCAVENGING PROPERTIES FOR THE TREATMENT OF AKI (TABLE 1)

### 4.1 Inorganic nanoparticles with ROS‐scavenging properties

#### 4.1.1 Metal oxide nanoparticles

**FIGURE 8:**
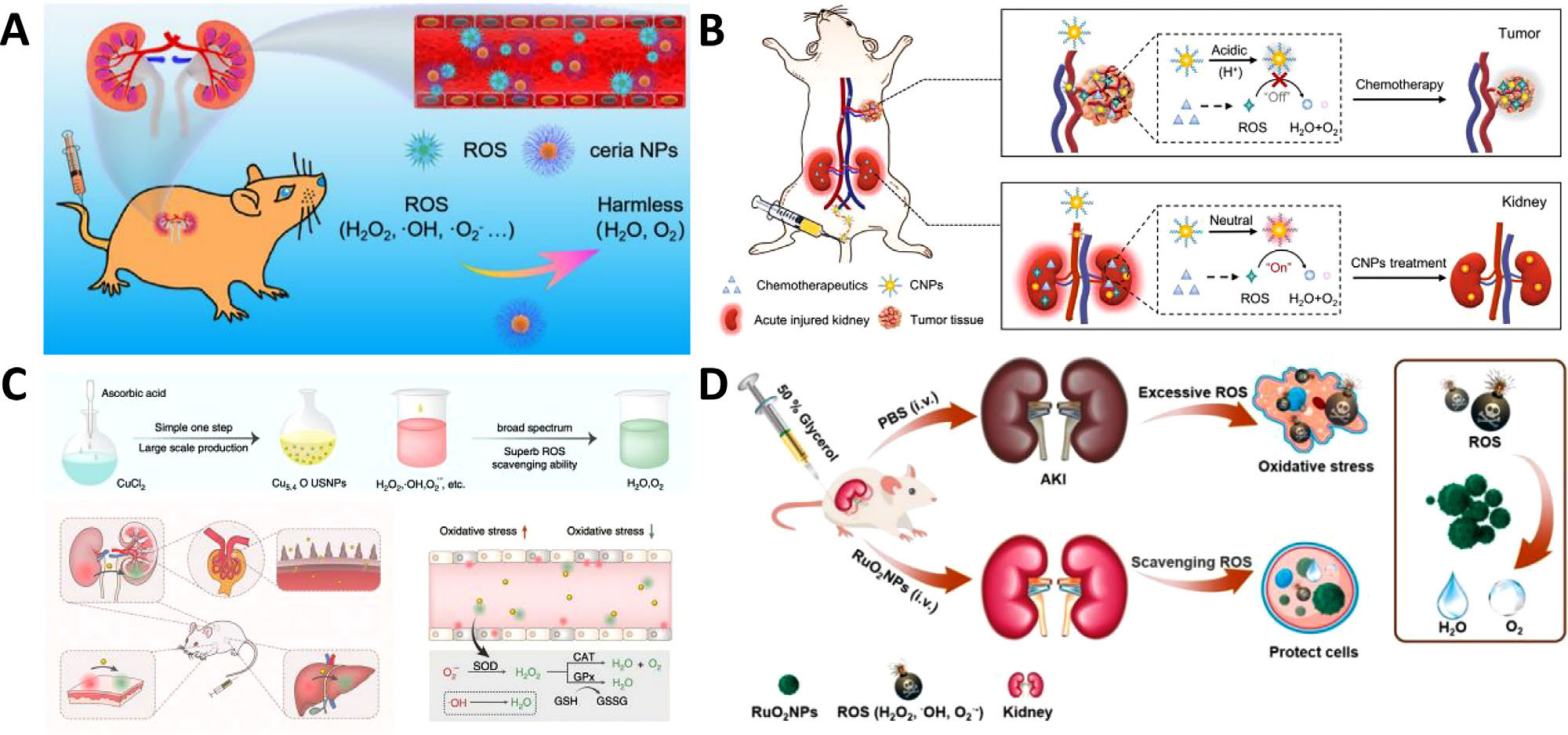
Inorganic metal oxide nanoparticles: (A) Ultrasmall citric acid modified ceria nanozymes with preferential kidney uptake for AKI alleviation through the elimination of excessive reactive oxygen species (ROS). Reproduced with permission.^[^ ^51b^ ^]^ Copyright 2020, American Chemical Society. (B) Tuneable ceria nanoparticles with context‐dependent ROS modulating activity and ROS‐involved gene‐regulating capability. Reproduced under the terms of the CC‐BY license.^[^ ^52^ ^]^ Copyright 2021, Qinjie Weng et al. (C) Ultrasmall Cu_5.4_O nanoparticles with multi‐enzyme‐like activity to alleviate oxidative‐stress‐mediated diseases. Reproduced under the terms of the CC‐BY license.^[^ ^53^ ^]^ Copyright 2020, Tengfei Liu et al. (D) Ultrasmall RuO_2_ nanoparticles with ROS scavenging properties and specific renal accumulation. Reproduced with permission.^[^ ^54^ ^]^ Copyright 2020, American Chemical Society.

Metal oxides are traditional catalysts for peroxides, and thus, engineering metal oxides into nanozymes can result in promising ROS scavenging properties. Ceria nanoparticles have been shown to have multienzyme‐like activities and the ability to eliminate a broad‐spectrum of ROS, and they have also been engineered into ceria nanozymes that can specifically target the GFB. Ceria nanoparticles can scavenge H_2_O_2_ through reversibly binding oxygen and shifting between the oxidized state (Ce^4+^) and the reduced state (Ce^3+^). Additionally, ceria nanoparticles can accept electrons from radicals and active reactant cytochrome c (Cyt c), indicating the multi‐enzyme activities against various ROS. An in vitro experiment demonstrated that the ceria nanozyme could decompose a significant portion of ROS even at low concentrations. However, whether renal accumulations could reach this concentration and whether the ROS scavenging ability stays intact in vivo are still uncertain (Figure 8A).^[^ ^51^ ^]^

To obtain more specific ROS‐neutralizing capabilities, Weng et al. engineered tuneable ceria nanoparticles to alleviate renal ROS levels, while maintaining chemotherapeutic efficacy. These nanodrugs demonstrated specific ROS scavenging activity in the renal cortex and the ability to regulate ROS‐involved genes. The tuneable ceria nanoparticles become inert due to excessive H^+^ in an acid tumour environment, allowing chemotherapy‐induced ROS to kill tumour cells (Figure 8B).^[^ ^52^ ^]^

The synthesizing processes for most nanodrugs are complicated, and have subsequent knock‐on effects for mass production methods. However, one study has reported a simple one‐step process to manufacture Cu_5.4_O ultrasmall nanoparticles that can considerably neutralize ROS. The ROS scavenging activities originate from Cu_5.4_O rather than the surface‐coated reductive materials (such as L‐ascorbic acid). Results showed that these particles could alleviate broad ROS‐related diseases, indicating poor specification. Thus, this kind of nanomedicine may not be suitable for chemotherapy‐induced AKI since the non‐specific activity would reduce the efficacy of the chemotherapy (Figure 8C).^[^ ^53^ ^]^

Liu et al. developed ultrasmall RuO_2_ nanoparticles (≈2 nm), which have considerable ROS‐eliminating abilities and low biological toxicity. Although the mechanisms are unclear, this metal oxide mimics the reaction of CAT and other enzymes, and the ultrasmall particle size allows them to be easily excreted. However, these high‐efficiency‐low‐toxicity nanoparticles also exhibit non‐specific biodistributions, which can result in global ROS alleviation, and will thus also affect chemotherapy (Figure 8D).^[^ ^54^ ^]^

#### 4.1.2 Noble metal nanoparticles

**FIGURE 9:**
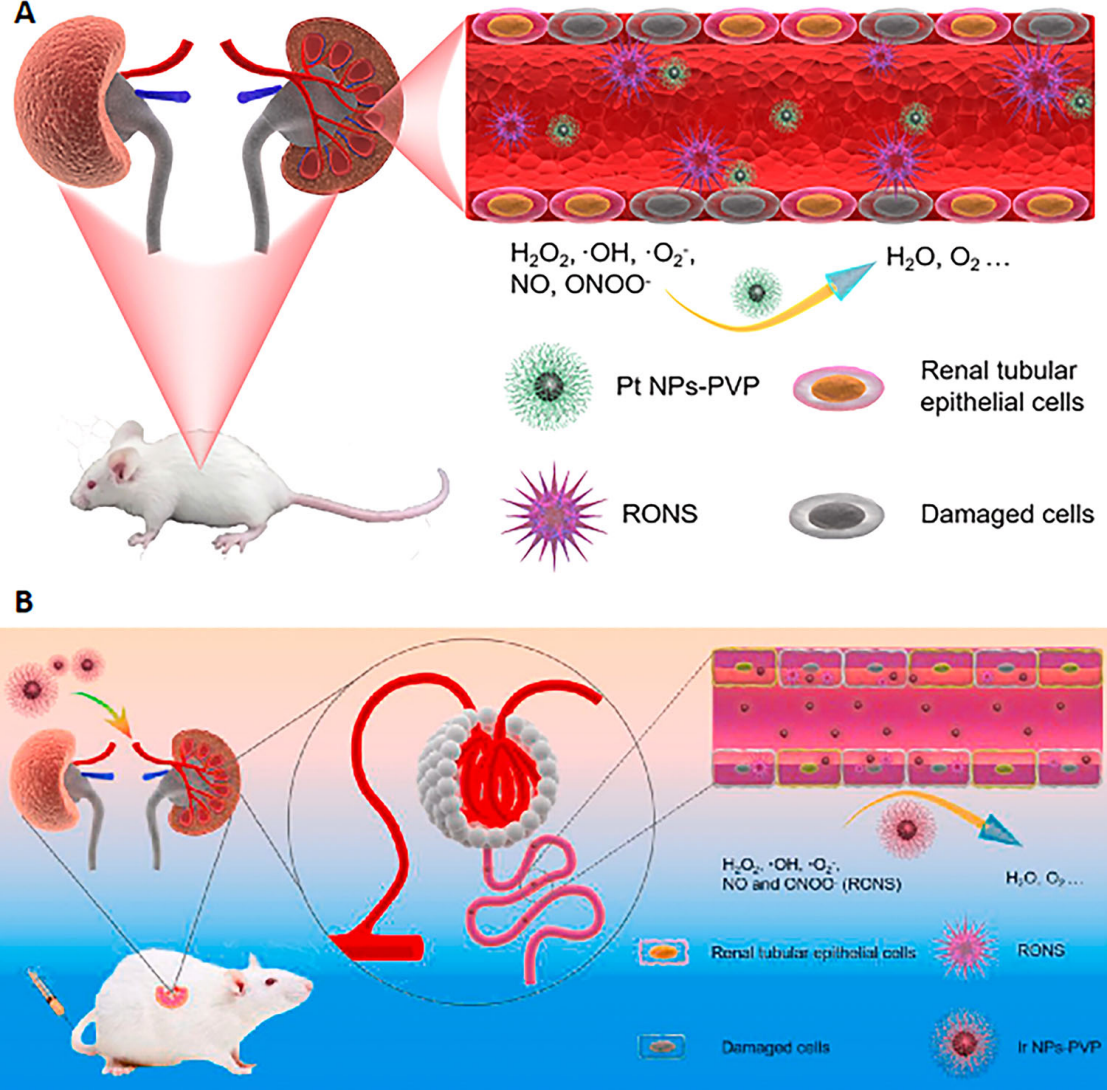
Inorganic noble metal nanoparticles: (A) Ultrasmall polyvinylpyrrolidone‐coated platinum nanoparticles exhibiting preferential kidney uptake and excellent antioxidative activity. Reproduced with permission.^[^ ^55^ ^]^ Copyright 2020, Elsevier. (B) Ultrasmall polyvinylpyrrolidone‐coated iridium nanoparticles with similar bioactivity indicate that specific renal accumulation and reactive oxygen species scavenging attributes may be shared among the noble metal family. Reproduced with permission.^[^ ^56^ ^]^ Copyright 2021, Elsevier.

Noble metals have several attractive attributes, such as stability, coordination ability, and low toxicity, while particles at the nanoscale can exhibit unique multi‐enzyme‐like activity. The combination of the two noble metal nanoparticles can thus have desirable properties. In recent years, platinum, iridium, and other noble metal nanomaterials have shown multi‐enzyme activity and excellent ROS scavenging capabilities. Subsequently, an ultrasmall PVP‐coated platinum nanoparticle (Pt NPs‐PVP, ≈1.5 nm) was developed to neutralize renal reactive oxygen/nitrogen species (Figure 9A). Pt NPs‐PVP can accept electrons from Cyt c without reliance on dissolved oxygen, demonstrating radical scavenging capability. The nanoparticle demonstrated preferential renal uptake and relatively low biotoxicity due to rapid excretion. The study presented a nanodrug that is promising for clinical AKI treatments and relatively easy to mass produce. Thus, engineering noble metals into nanoscale particles could be favourable for further research.^[^ ^55^ ^]^ The substitution of iridium for platinum and construction of Ir NPs‐PVP (≈3 nm) were also found to have similar in vivo activities, indicating that these attributes may belong to the noble metal family (Figure 9B). The ROS scavenging mechanism is also very similar to Pt NPs‐PVP, which can accept electrons independent of dissolved oxygen.^[^ ^56^ ^]^

#### 4.1.3 Two‐dimensional nanostructures

**FIGURE 10:**
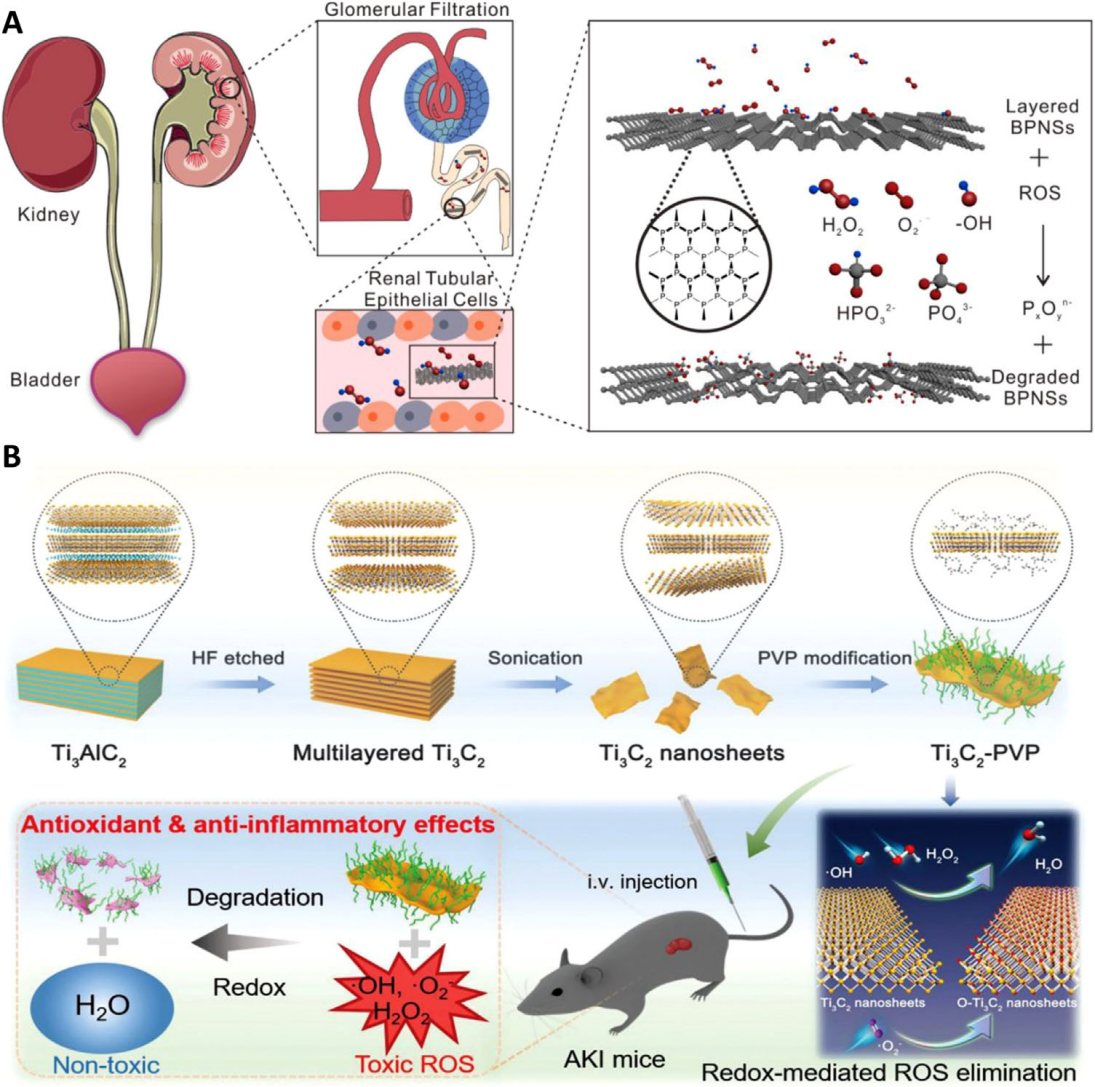
Two‐dimensional nanostructures: (A) Layered black phosphorus nanosheets could specifically target the renal area and eliminate renal reactive oxygen species (ROS). Additionally, the nanosheets would degrade into harmless materials after reacting with ROS. Reproduced with permission.^[^ ^58^ ^]^ Copyright 2020, American Chemical Society. (B) The polyvinylpyrrolidone‐modified Ti_3_C_2_ nanosheets have properties similar to black phosphorus‐based nanosheets: ROS reactive and degradable after reaction with ROS. Although the biodistribution of this nanosheet is unclear, it presents a novel concept for nanostructure engineering. Reproduced under the terms of the CC‐BY license.^[^ ^59^ ^]^ Copyright 2021, Xing Zhao et al.

Previous research shows that nanomaterials with two‐dimensional structures have a broad range of medical applications due to their complexity compared to simple nanodots. Black phosphorus nanosheets have shown promising potential in biomedical applications and have remarkable applications in photothermal therapy due to their unique attributes^[^ ^57^ ^]^ (Figure 10A). Additionally, as one of the most bio‐reactive materials, black phosphorus nanosheets can actively scavenge ROS. The molecular structure empowers black phosphorus nanosheets with excellent ROS‐consuming capability. As an elemental phosphorus, the nanosheets have weak van der Waals forces due to the composition with single‐ or few‐layer black phosphorus, thus holding great chemical relatives. The layered structure of black phosphorus nanosheets and elemental state enable rapid electron transfer and easy oxidative reaction to form P─O bonds, which demonstrates the powerful capability of ROS scavenging. The nanosheet also has preferential renal uptake, making it an effective nanomaterial to alleviate oxidative stress‐induced cellular apoptosis. Although enjoying many promising attributes, the biotoxicity of black phosphorus nanosheets is not thoroughly understood. After reacting with ROS, some nanosheets can degrade into benign phosphorus oxides, but those that are unreacted could cause significant adverse effects.^[^ ^58^ ^]^

Apart from phosphorus, Zhao et al. have developed ultrathin Ti_3_C_2_‐PVP nanosheets with excellent biocompatibility and significant ROS scavenging capabilities (Figure 10B). Ti_3_C_2_‐PVP consumes ROS through the redox reaction between ROS and Ti_3_C_2_, and the mechanism is verified by density functional theory calculation. The nanosheets are ≈200 nm in lateral dimension and were surface‐modified with PVP to improve colloidal stability. In this study, the team thoroughly examined the laboratory properties of the nanosheets and confirmed that they could considerably alleviate AKI in animal experiments. However, the biodistribution is not thoroughly examined, and the biotoxicity remains undetermined.^[^ ^59^ ^]^ Nevertheless, the favourable attributes indicate that engineering higher‐dimensional complex nanostructures could aid in developing effectively‐targeting medicines (See Table 1).

#### 4.1.4 Coordination complex nanoparticles

**FIGURE 11:**
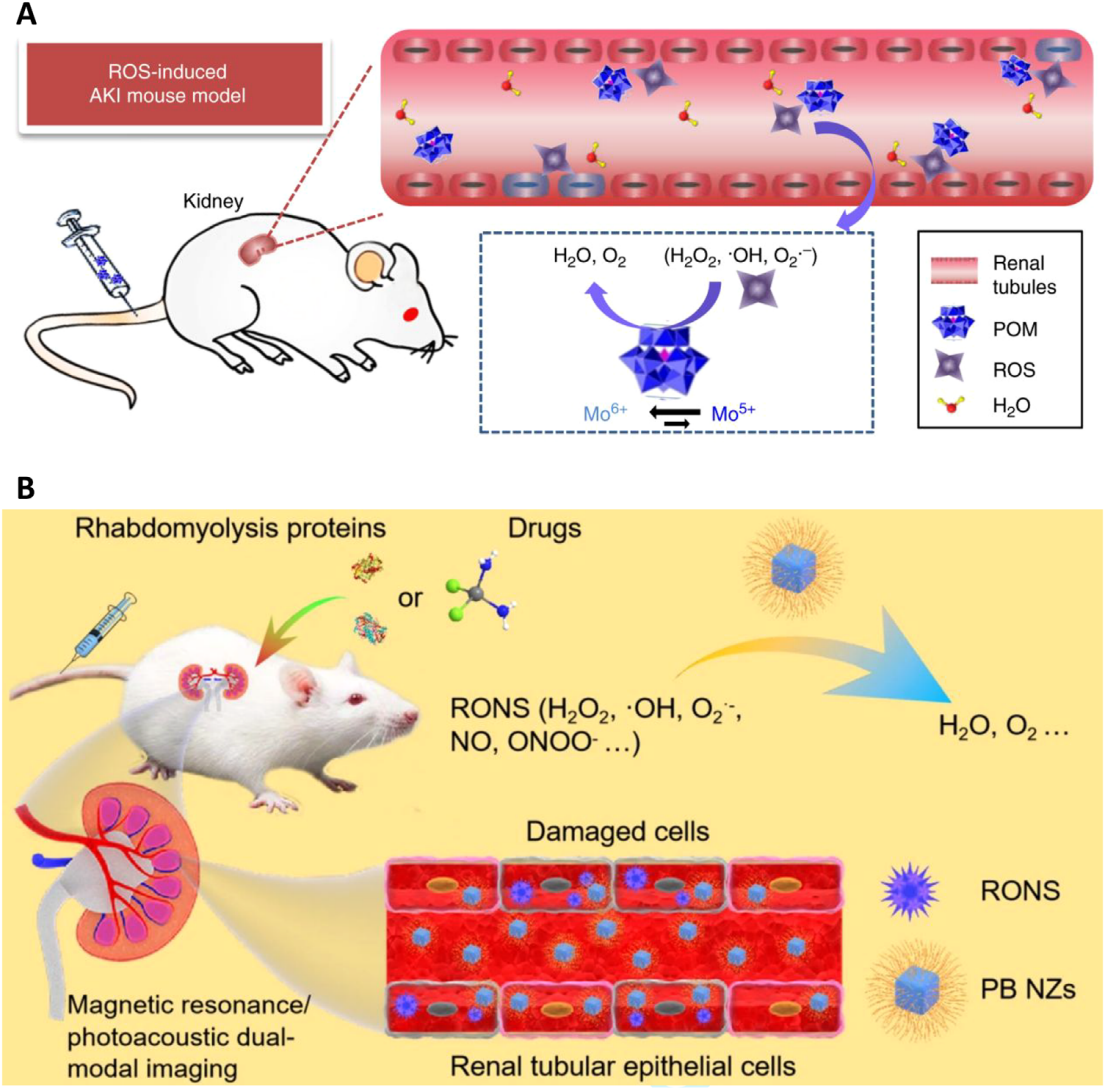
Coordination complex nanoparticles: (A) Molybdenum‐based polyoxometalate nanoclusters with specified renal accumulation and excellent ROS alleviating capabilities. Reproduced under the terms of the CC‐BY license.^[^ ^29^ ^]^ Copyright 2018, Dalong Ni et al. (B) Ultrasmall Prussian blue nanozymes with rapid renal aggregation to ameliorate AKI by reducing excessive reactive oxygen/nitrogen species. Reproduced under the terms of the CC‐BY license.^[^ ^60^ ^]^ Copyright 2021, Dong‐Yang Zhang et al.

In coordination complexes, central atoms, mostly metal ions, can have minor oxidative capabilities, empowering them with a CAT‐like ability. Ligands, on the other hand, provide the entire structure with more functionality and can be engineered for specific targeting. Using a simple one‐pot method, the researchers have synthesized molybdenum‐based polyoxometalate (POM) nanoclusters with considerable ROS‐scavenging abilities. The ROS scavenging mechanism of POM is similar to that of ceria nanoparticles, as the Mo^5+^ is reversibly oxidized to Mo^6+^ when eliminating the ROS. Both cell experiments and in vivo models demonstrate effective ROS neutralization and renal cell protection abilities. This ROS alleviation is attributed to the easily changed valency between Mo^5+^ and Mo^6+^, as the Mo ion has a low redox potential. In addition, due to the small size of the nanocluster (<10 nm), it can successfully pass through the GFB and shows high levels of accumulation in the renal area (Figure 11A).^[^ ^29^ ^]^

Prussian blue is a widely used painting material, and it is surprising to find its medical application. The primary component of Prussian blue is Fe(CN)_6_, which has a CAT‐like ability. This property led Zhang et al. to develop Prussian blue nanozymes (≈4.5 nm), which exhibit rapid renal accumulation and in vivo multi‐enzyme activity. However, the ROS scavenging mechanism is not investigated in the study. Furthermore, the nanozymes demonstrated superior AKI treatment efficacy in animal experiments. This study suggested that additional engineering of traditional materials could lead to exciting and surprising developments in nanomedicine (Figure 11B).^[^ ^60^ ^]^

### 4.2 Organic nanoparticles (or metal ion doped organic nanoparticles) with ROS‐scavenging properties

#### 4.2.1 Organic nanoparticles

Inorganic nanomaterials have excellent ROS scavenging and renal accumulation abilities. However, nearly all inorganic materials have considerable biotoxicity, especially metal compounds. On the other hand, organic nanoparticles could significantly reduce in vivo toxicity if well‐designed. For instance, many endogenous compounds in the human body have ROS‐scavenging activity and thus can be further engineered into antioxidant nanoparticles. This kind of endogenous‐origin material may have lower levels of in vivo interactions with macrophages and other immune cells, thus increasing the chance of survival. Additionally, organic materials can be engineered into complex and functional nano frameworks, enabling further construction, such as metal doping (Table 2).

**TABLE 1:** Engineered nanodrug in the treatment of AKI. Green: Inorganic nanoparticles with properties of scavenging reactive oxygen/nitrogen species (RONS). Blue: Organic nanoparticles with RONS scavenging properties. Orange: DNA‐based nanoparticles with RONS scavenging properties. Yellow: Engineered nanocarrier loaded with antioxidants (H_2_O_2_: Hydrogen peroxide, •OH: Hydroxyl radicals, •O_2_^−^: Superoxide radicals, DPPH•: 1,1‐diphenyl‐2‐picrylhydrazyl radical), ABTS•: 2,2′‐azino‐bis(3‐ethylbenzothiazoline‐6‐sulfonic acid), ONOO^−^: Peroxynitrite, NO: Nitric oxide.).

Engineered nanodrugs	Composition	Active drug compound	Size	Shape	Electrical charge	Surface modification	Type of RONS	Ref.
Multi‐enzyme mimetic ultrasmall iridium nanozymes	Iridium trichloride hydrate, PVP	Iridium	Hydrodynamic size: 1.5 nm	Spherical	−4 mV in PBS	PVP	H_2_O_2_, •OH, •O_2_^−^, DPPH•, ABTS•, ONOO^−^ and NO	^[^ ^56^ ^]^
Ultrasmall RuO_2_ nanoparticles	RuO_2_	RuO_2_	Average size: 2 nm	Spherical	Not mentioned	PVP	H_2_O_2_, •OH and •O_2_^−^	^[^ ^54^ ^]^
Ultrasmall PVP‐platinum nanoparticles	Platinum, PVP	platinum	≈3 nm	Spherical	Not mentioned	PVP	H_2_O_2_, •OH, •O_2_^−^, DPPH•, ABTS•, ONOO^−^ and NO	^[^ ^55^ ^]^
Black phosphorus quantum dots	Black phosphorus	Black phosphorus	≈3.5 nm	Spherical	−6 mV in water	None	H_2_O_2_, •OH, •O2^−^ and ABTS•	^[^ ^32^ ^]^
Ultrasmall Prussian blue nanozymes	Prussian blue, biopolymer chitosan	Fe^3+^ and Fe^2+^	≈4.5 nm	Spherical	Not mentioned	Biopolymer chitosan	H_2_O_2_, •OH, •O_2_^−^, DPPH•, ABTS•, ONOO^−^ and NO	^[^ ^60^ ^]^
Ultrasmall Cu_5.4_O nanoparticles	Cu_5.4_O, l‐ascorbic acid	Cu_5.4_O	Hydrodynamic size: <5.5 nm	Spherical	Not mentioned	None	H_2_O_2_, •OH, •O_2_^−^, ABTS•, ONOO^−^ and NO	^[^ ^53^ ^]^
Ultrasmall KCa(H_2_O)_2_[Fe^III^(CN)_6_]·H_2_O nanoparticles	Fe^III^[(CN)_6_]^3−^, PVP, CaCl_2_	KCa(H_2_O)_2_[Fe^III^(CN)_6_]·H_2_O	hydrodynamic size: ≈6 nm	Spherical	−7.27 mV in water	PVP	H_2_O_2_, •OH, •O_2_^−^, DPPH•, ONOO^−^ and NO	^[^ ^67^ ^]^
Ceria Nanoparticles	Ceria, PEG	A mixture of Ce^3+^ and Ce^4+^	Hydrodynamic size: ≈9.7 nm	Spherical	−20.4 mV in water	DSPE‐PEG_2K_	H_2_O_2_, •OH, •O_2_^−^ and ABTS•	^[^ ^51^, ^52^, ^80^ ^]^
Molybdenum‐based polyoxometalate nanocluster	(NH_4_)_6_Mo_7_O_24_·4H_2_O and NaH_2_PO_4_	Mo ion	≈1 nm (hydrated size: <10 nm)	Spherical	Not mentioned	None	H_2_O_2_, •OH, •O_2_^−^ and ABTS•	^[^ ^29^ ^]^
Ultrathin Ti_3_C_2_‐PVP nanosheet	Ti_3_C_2_	Ti_3_C_2_	Lateral dimension ≈200 nm	2D nanosheet	Approximately neutral	PVP	H_2_O_2_, •OH, •O_2_^−^ and ABTS•	^[^ ^59^ ^]^
Black phosphorus nanosheet	Black phosphorus	Black phosphorus	Diameter ≈225 nm Thickness 3.8–4.5 nm	2D nanosheet	−22.5 mV in water	None	H_2_O_2_, •OH, •O_2_^−^ and ABTS•	^[^ ^58^ ^]^
Selenium‐doped carbon quantum dot	Selenium, carbon quantum dots	Selenium	Diameter: 40 nm; height: 2 nm	Spherical	−31 mV	None	H_2_O_2_, •OH, •O_2_^−^ and ABTS•	^[^ ^81^ ^]^
1‐dodecanethiol stabilized Mn_3_O_4_ (dMn_3_O_4_) nanoparticle inside ROS‐sensitive nanomicelles	1‐dodecanethiol Mn_3_O_4_, ROS‐sensitive nanomicelles	Mn_3_O_4_	dMn_3_O_4_: ≈18 nm Nanomicelles: ≈400 nm	Spherical	Nanomicelles: −5.3 mV	None	H_2_O_2_	^[^ ^65^ ^]^
Ultrasmall Mn^2+^‐chelated melanin nanoparticle incorporated with polyethylene glycol (MMPP)	Melanin, Mn^2+^, PVP, Polyethylene glycol (PEG)	Melanin	Hydrodynamic size: 4.5 nm	Spherical	−7.8 ± 1.0 mV	PVP and HS‐PEG	H_2_O_2_, •OH, •O_2_^−^ and ABTS•	^[^ ^31^ ^]^
Self‐assembled ultrasmall nanodot	Iron ion, gallic Acid, PVP	Iron ion, gallic acid	Hydrodynamic size: 3.5 nm	Spherical	Not mentioned	PVP	H_2_O_2_, •OH, •O_2_^−^, DPPH•, ONOO^−^ and NO	^[^ ^30^ ^]^
Fe–curcumin coordination polymer nanodot	Fe–curcumin	Fe–curcumin	<10 nm	Spherical	Not mentioned	None	H_2_O_2_, •OH, •O_2_^−^, DPPH• and ABTS•	^[^ ^66^ ^]^
Hyaluronic acid coated bilirubin nanoparticle	ε‐polylysine‐bilirubin, hyaluronic acids	bilirubin	Hydrodynamic diameter: 226.9 ± 4.5 nm	Spherical	−23.9 ± 0.2 mV	Hyaluronic acid	H_2_O_2_	^[^ ^61^ ^]^
PVP‐curcumin nanoparticles	PVP, curcumin	Curcumin	5∼8 nm	Spherical	Not mentioned	None	Not mentioned	^[^ ^82^ ^]^
Polydopamine wrapped manganese ferrite nanoparticles	Polydopamine, manganese ferrite	Polydopamine, manganese ferrite	≈140 nm	Spherical	−25 mV	Polydopamine	H_2_O_2_	^[^ ^64a^ ^]^
DNA origami nanostructures	DNA	DNA	Rectangular: 90 nm×60 nm; triangular: 120 nm per edge; tubular: 400 nm long	Rectangular, triangular, and tubular	Rectangular: −2.8 mV, triangular: −3.4 mV, tubular: −1.6 mV	None	H_2_O_2_, •OH, •O_2_^−^ and ABTS•	^[^ ^40^ ^]^
DNA nanodevice	Rectangular DON, anticomplement component 5a aptamers	DNA, anticomplement component 5a aptamers	90 nm×60 nm	Rectangular	Not mentioned	Anticomplement component 5a aptamers	H_2_O_2_, •OH, •O_2_^−^ and ABTS•	^[^ ^70^ ^]^
Tetrahedral DNA nanostructures	DNA	DNA	Diameter: 18.79 nm	Tetrahedral	Not mentioned	None	Not mentioned	^[^ ^83^ ^]^
Tetrahedral framework nucleic acids	DNA	DNA	11.7 nm	Tetrahedral	Slightly negative	None	H_2_O_2_	^[^ ^71^ ^]^
Oltipraz‐loaded poly (lactic‐*co*‐glycolic acid) (PLGA) nanoparticle	PLGA, Oltipraz	Oltipraz	Hydrodynamic size: 100 nm	Spherical	−20 ± 6.7 mV	None	Not mentioned	^[^ ^33^ ^]^
Quercetin polymeric nanoparticle	PEG conjugated polyethyleneimine nanoparticles, quercetin	Quercetin	20.92 nm	Spherical	Not mentioned	None	Not mentioned	^[^ ^84^ ^]^
Ultrasmall gold nanocluster capped with *N*‐acetylcysteine	Gold nanocluster, *N*‐acetylcysteine	*N*‐acetylcysteine	1–2 nm	Spherical	−47.8 ± 3.5 mV	*N*‐acetylcysteine	H_2_O_2_, •OH, •O_2_^−^ and ABTS•	^[^ ^79^ ^]^
SS‐31 delivering, pH‐responsive, AKI kidney targeting nanopolyplexes	Anionic hyaluronic acid, cationic chitosan, SS‐31	SS‐31	Hydrodynamic Size: 53 ± 0.17 nm	Spherical	−19.6 ± 0.7 mV	Anionic hyaluronic acid	H_2_O_2_	^[^ ^49^ ^]^
Modified ceria nanoparticle loaded with atorvastatin	Ceria, triphenylphosphine, mPEG‐TK‐PLGA, atorvastatin	Ce^3+^ and Ce^4+^, atorvastatin	Hydrodynamic Size: 43.1 ± 7.50 nm	Spherical	−4.45 ± 0.751 mV	mPEG‐TK‐PLGA	H_2_O_2_	^[^ ^78^ ^]^

**TABLE 2:** Comparison of advantages and disadvantages of organic and inorganic nanoparticles based engineered nanodrugs in treating AKI.

Inorganic nanoparticles‐based engineered nanodrugs		Organic nanoparticles‐based engineered nanodrugs	
Advantages	Disadvantages	Advantages	Disadvantages
More stable Clearer mechanisms Easier to synthesize and Smaller and better targeting GFB	Generally higher biotoxicity More limited functionalities	Lower biotoxicity More complex structures enable more functionalities	More difficult to synthesize More complicated mechanisms Less stable both in vivo and in vitro Less specific biodistribution

**FIGURE 12:**
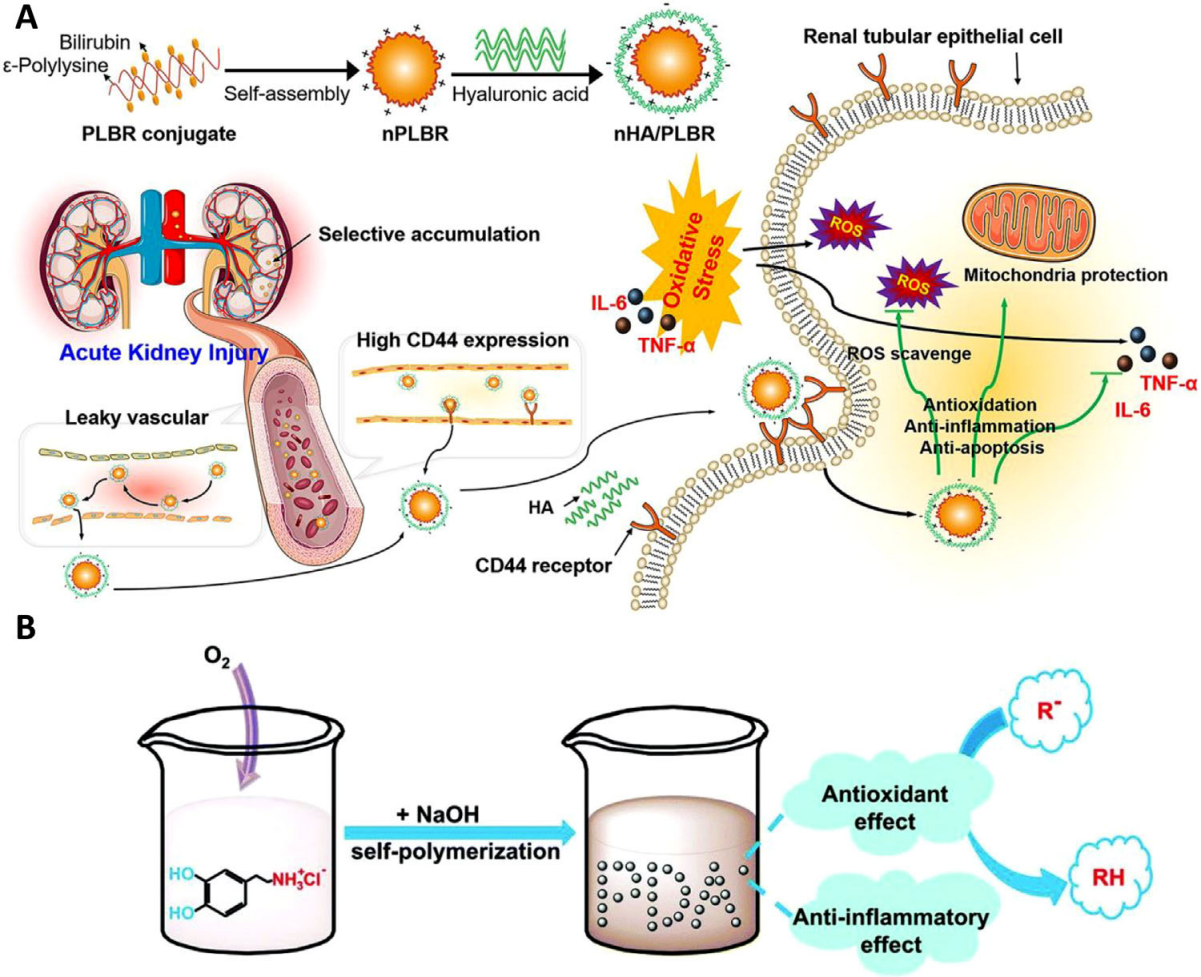
Organic nanoparticles: (A) Organic nanoparticles: hyaluronic acid coated ε‐polylysine‐bilirubin conjugate nanoparticles that can selectively accumulate in injured kidneys and alleviate ROS‐induced renal damage. Reproduced with permission.^[^ ^61^ ^]^ Copyright 2021, Elsevier. (B) The synthesis process of melanin‐similar polydopamine nanoparticles with low biotoxicity, antioxidant effect, and anti‐inflammatory capability. Reproduced with permission.^[^ ^62^ ^]^ Copyright 2018, Royal Society of Chemistry.

Bilirubin is an internal compound with ROS‐eliminating abilities. Zhi‐Wei Huang et al. have developed a hyaluronic acid‐coated ε‐polylysine‐bilirubin conjugate nanoparticle with specific renal accumulation and oxidation/inflammatory alleviation ability. Bilirubin has considerable in vivo ROS scavenging activities. Forming bilirubin into nanoparticles above drastically improved the ROS eliminating capability (Figure 12A). In vitro experiment results demonstrated high biostability, good biocompatibility, significant antioxidation, and anti‐apoptotic effects. Additionally, in vivo studies confirmed renal‐specific biodistribution and cell protection effects. This study shows that endogenous compounds may be suitable for developing potential nanomedicines.^[^ ^61^ ^]^

In addition, it is usually beneficial to construct particles with properties similar to those of natural particles. Scientists developed polydopamine nanoparticles, whose structures and chemical attributes were similar to those of melanin, a natural bio‐polymer (Figure 12B). The particles demonstrated considerable antioxidative capabilities, both in vivo and in vitro. The nanoparticles not only act as a reducing agent reacting with ROS, but also have catalyst‐like activities to decompose H_2_O_2_. The low biotoxicity and effective anti‐inflammatory abilities further revealed the potential of organic nanodrugs.^[^ ^62^ ^]^

#### 4.2.2 Metal ion doped organic nanoparticles

Many organic compounds, especially endogenous ones, have desirable attributes and can thus be engineered into effective nanodrugs. However, pure organic nanoparticles may be hard to synthesize and purify, thus making them unsuitable for mass production. However, metal ion doped organic nanoparticles might resolve this problem and lead to further application.

**FIGURE 13:**
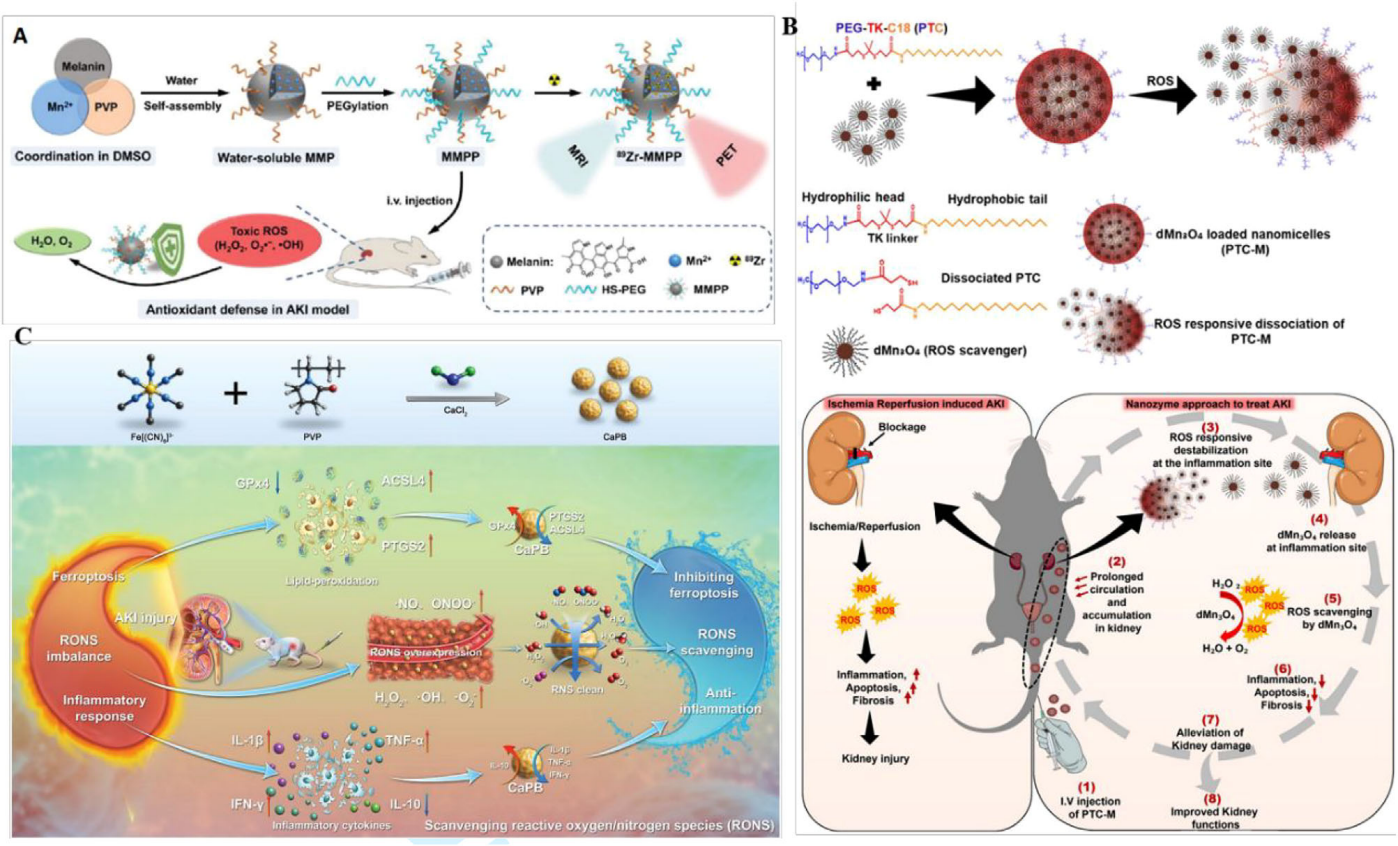
Metal ion doped organic nanoparticles: (A) Ultrasmall Mn^2+^‐chelated melanin coated with PVP with specific renal accumulation and antioxidative ability. Reproduced with permission.^[^ ^31^ ^]^ Copyright 2019, Wiley. (B) 1‐dodecanethiol stabilized Mn_3_O_4_ capped in ROS‐sensitive nanomicelles, resulting in satisfactory biodistribution and oxidative alleviation. Reproduced under the terms of the CC‐BY license.^[^ ^65^ ^]^ Copyright 2022, Hong Sang Choi et al. (C) CaPB nanozymes, multi‐enzyme mimetics, could scavenge RO/NSs for AKI treatment via the inhibition of ferroptosis and anti‐inflammation. Reproduced with permission.^[^ ^67^ ^]^ Copyright 2021, Wiley.

A research team has also engineered ultrasmall Mn^2+^‐chelated melanin via coordination and self‐assembly synthesis and incorporated it with polyethylene glycol to improve in vivo stability (Figure 13A). As a critical endogenous biopolymer, melanin has excellent biocompatibility and biodegradation in biomedical applications, with nearly no side effects.^[^ ^31^, ^63^ ^]^ Additionally, Mn^2+^ is a traditional inorganic catalyst for peroxide, and combining this material with melanin, which is also an antioxidant, can enhance this activity. The results of in vitro experiments demonstrate multi‐antioxidative activities, and those of in vivo experiments show low levels of biotoxicity and moderate renal protection in the glycerol‐induced AKI model despite non‐specific biodistribution. Although the non‐specific biodistribution considerably inhibits clinical applications, the engineering results are encouraging and indicate that this method should be the focus of further research.^[^ ^31^ ^]^

Recently, researchers have reported a self‐assembled ultra‐small nanodot that is approximately 3.5 nm and composed of an iron ion, gallic acid, and PVP. Gallic acid is a polyphenol extracted from natural plants, which has excellent antioxidant properties, while iron is a transition metal with excellent ROS‐scavenging activity. Combining these two attributes endows the nanodot with a broad‐spectrum ROS‐scavenging ability. The nanodots exhibit SOD activities and thus can neutralize ROS. In vitro, these nanodots protect cells against H_2_O_2_‐induced apoptosis, while in vivo, the nanoparticles aggregate preferentially in the renal area and are quickly excreted in the urine. Furthermore, the nanodot demonstrates enhanced biocompatibility and biodegradability, magnifying the therapeutic effects compared to traditional small‐molecule drugs.^[^ ^30^ ^]^ This study confirms that combining metal ions and organic compounds with desirable attributes has a promising future in novel nanodrug engineering.

In addition to the molecules that only scavenge ROS to alleviate oxidative stress, polydopamine‐wrapped manganese ferrite nanoparticles were developed, which could produce O_2_ by eliminating ROS. These constructed nanoparticles can react with ROS and will transform into oxidation products, including pyrrole‐2,3‐dicarboxylic acid. The generated oxygen can convert pro‐inflammatory M1‐type macrophages into anti‐inflammatory M2‐type macrophages, further relieving oxidative stress in the renal area. By employing the product of ROS elimination as a regulator for macrophages and explaining the mechanism of renal protection, this research presents us with a novel perspective for nanomedicine engineering.^[^ ^64^ ^]^

In addition, a scientific team encapsulated 1‐dodecanethiol‐stabilized Mn_3_O_4_ (dMn_3_O_4_) in ROS‐sensitive nanomicelles (Figure 13B). The active compound, dMn_3_O_4_, is approximately 18 nm, while the full nanomicelles are approximately 400 nm. The enclosure collapses with oxidative states, releasing ROS scavenging dMn_3_O_4_ to alleviate renal ROS levels. This captured drug delivery system could help to deliver effective nanodrugs to targeted sites, reducing potential adverse effects. The nanomicelles can reduce apoptosis by affecting the mitogen‐activated protein kinase signalling pathway. The Mn_3_O_4_ nanoparticles possess Mn^2+^ and Mn^3+^ and have the ability to decompose H_2_O_2_ to H_2_O and O_2_, thus preventing ROS‐mediated cell damage. However, the ex vivo fluorescence images show extensive liver uptake and relatively poor renal accumulation, which may lead to high levels of biotoxicity.^[^ ^65^ ^]^ Nevertheless, this study perceives that encapsulating nanodrugs in larger nano‐enclosures may result in desirable attributes.

Various natural products have antioxidative characteristics, such as curcumin, quercetin, gallic acid, and proanthocyanidin. However, mass clinical usage is hindered by poor stability in physiological environments. Thus, a research team synthesized ultrasmall coordination polymer nanodots based on natural products coordinated with Fe ions. The nanodots display superb antioxidative capabilities and have a superior survival rate.^[^ ^66^ ^]^ Consequently, engineering unstable antioxidants into coordination complexes could lead to stable and superior attributes.

Moreover, scientists have also proposed ultrasmall KCa(H_2_O)_2_[Fe^III^(CN)_6_]·H_2_O nanoparticles (CaPB). CaPB demonstrated effective ROS alleviation and ferroptosis inhibition capabilities. The CaPB nanoparticles exhibit excellent stability and broad high‐performance catalytic activities, including SOD, CAT, POD, and GPx‐like activities. Moreover, the specific accumulation in the renal area regulates the abnormal expression of inflammatory factors. These desired properties help to rebalance the scales between ROS and antioxidants (Figure 13C).^[^ ^67^ ^]^

### 4.3 DNA‐based nanoparticles with ROS scavenging properties

In addition to the complex structures mentioned above, DNA also has excellent intrinsic properties, including low toxicity, low immunogenicity, and high levels of biological stability.^[^ ^68^ ^]^ Since DNA is sensitive to ROS, engineering it into ROS scavenging nanostructures could be a wise choice.

**FIGURE 14:**
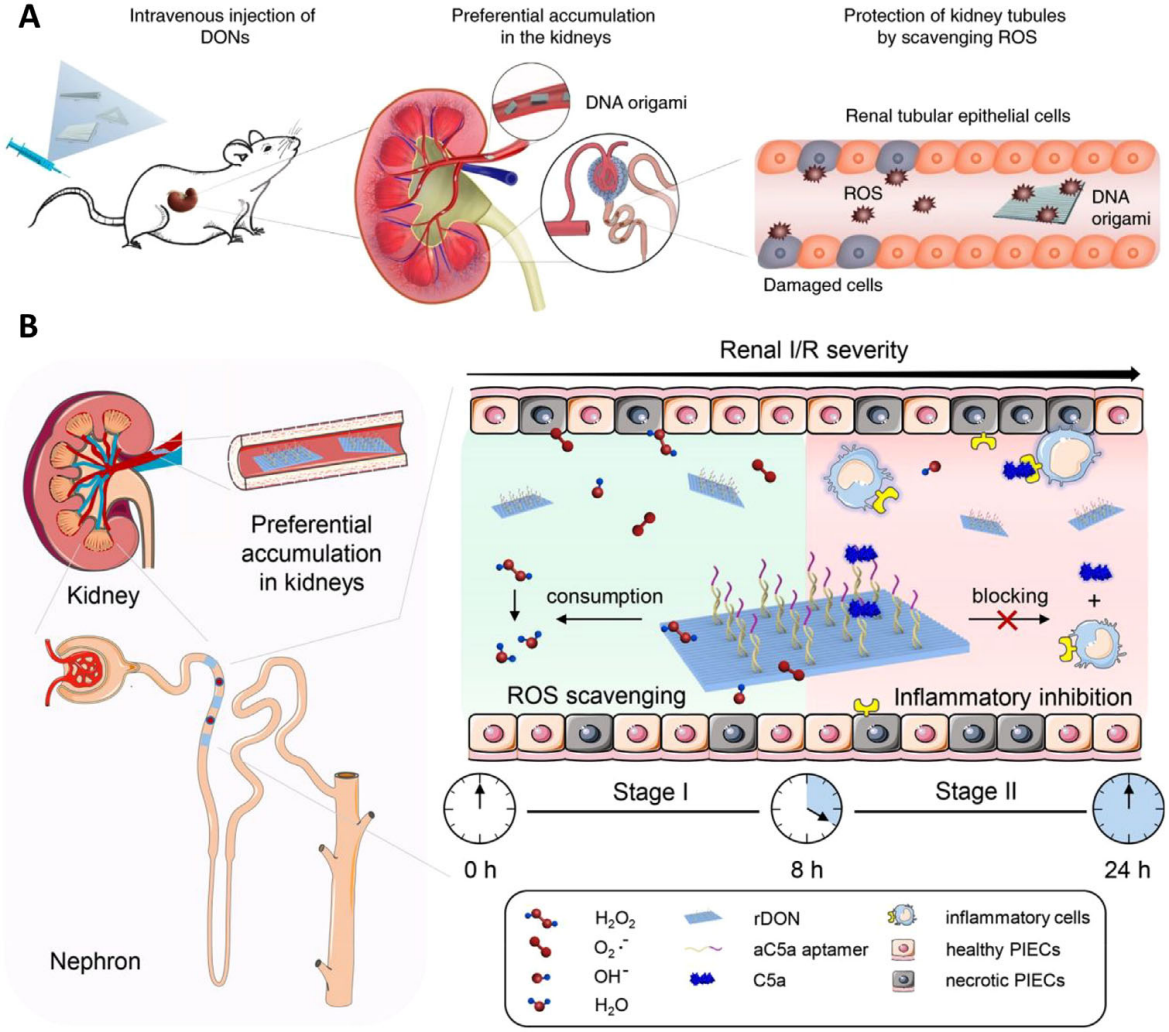
DNA‐based nanoparticles: (A) Three DNA origami nanostructures that display specific renal uptake and ROS eliminating abilities respectively, thus suitable for use in AKI treatments. Reproduced with permission.^[^ ^40^ ^]^ Copyright 2018, Springer Nature. (B) Rectangular DNA origami based nanodevice loaded with anticomplement component 5a, resulting in inflammatory inhibition. Reproduced with permission.^[^ ^70^ ^]^ Copyright 2021, American Chemical Society.

In previous studies, researchers proposed DNA origami nanostructures as a new type of nanomedicine to deliver small molecule drugs to tumour sites for tumour imaging and treatment.^[^ ^69^ ^]^ Jiang et al. took advantage of this attribute and engineered rectangular DNA origami nanostructures, which were found to aggregate in the renal area specifically and alleviate AKI.^[^ ^40^ ^]^ These DNA‐based nanostructures originate from M13 single‐stranded DNA, which has significant ROS scavenging properties. In vitro cell experiment results demonstrate that both single‐stranded DNA and DNA origami alleviate ROS stress, while the origami nanostructures endow the DNA with specific renal‐targeting abilities (Figure 14A).^[^ ^40^ ^]^ This research presents us with ingenious engineering strategies and demonstrates the potential of designing and engineering traditional molecules with desirable attributes into nanostructures.

Based on the rectangular DNA origami nanostructure, another research group synthesized sequential therapeutic nanodevices to scavenge ROS and suppress inflammatory responses effectively (Figure 14B). The scientists loaded anticomplement component 5a, which could block C5a in stage II, into a DNA structure, forming a sequential therapy.^[^ ^70^ ^]^ This inventive combination provides a dual‐target nanodrug and pushes the potential of nanostructures even further.

Furthermore, DNA structures have also been developed into 3D structures by constructing tetrahedral framework nucleic acids, which act as antioxidants in AKI treatments. The 3D nanostructures provide even more possibilities for nanodevice engineering. The cavity inside the tetrahedron could be used for molecule loading or other functions, which may result in surprising clinical effects.^[^ ^71^ ^]^

### 4.4 Engineering nanocarriers loaded with antioxidants

Nanocarriers have vast applications in the delivery and controlled release of drugs. Many studies have used nanomaterials to encapsulate small‐molecule drugs and macromolecules, such as nucleic acids, proteins, and peptides. Nanocarriers can change the drug's pharmacokinetics, such as the molecule's solubility.^[^ ^72^ ^]^ They can also target specific organs, tissues, or cells of the human body and release the carried drugs into the target site, obviating the side effects on physiological structures caused by off‐target delivery and high doses.^[^ ^15a^ ^]^ In addition, the timed, quick, controllable, and single‐dose release of drugs was found to achieve the best therapeutic results.^[^ ^73^ ^]^ Furthermore, nanocarriers can protect encapsulated drugs from harsh in vivo environments, improving their stability and efficacy.^[^ ^15^, ^17^, ^74^ ^]^ To ensure superior therapeutic efficiency, the nanocarriers need a relatively extended circulatory half‐life and specific drug‐releasing abilities, which are influenced by biocompatibility, monodispersity, and conditional responsibility (such as pH or temperature). Scientists have come up with innovative strategies to achieve the maximum in vivo functionalities, including organic‐polymer‐based, inorganic‐material‐based, and bio‐mimics, which show excellent insights to learn from nature to develop impressively complex particles.^[^ ^75^ ^]^ However, this technique has not been applied in AKI treatment, but it will indeed become the most influential trend in nanodrug engineering.

#### 4.4.1 Engineered nanocarriers based on organic polymers

A poly lactic‐co‐glycolic acid nanocarrier that is ≈100 nm and can selectively aggregate in injured kidneys was developed. The researchers used the small molecule Oltipraz as the carried molecule, which alleviates oxidative stress and has significant potential for renal treatment.^[^ ^76^ ^]^ To extend the in vivo survival time, the researchers loaded Oltipraz into the nanocarrier, resulting in prolonged specific renal aggregation and controlled drug release. Additionally, during the in vitro cell experiments, when compared with Oltipraz alone, the nanocarrier loaded with Oltipraz displayed superior antioxidation ability, higher SOD activity, and significantly improved cell viability. Furthermore, the nanocarrier loaded with Oltipraz demonstrated a considerable renal protection effect during in vivo experiments (Figure 4).^[^ ^33^ ^]^ This study showed that using a nanocarrier to precisely deliver small molecule drugs could significantly improve overall efficacy. However, as the size of the nanocarrier in the study may have been too large for rapid excretion, it resulted in prolonged renal accumulation, which may lead to severe adverse effects. Thus, further biotoxicity examinations are required for its potential clinical application.

**FIGURE 15:**
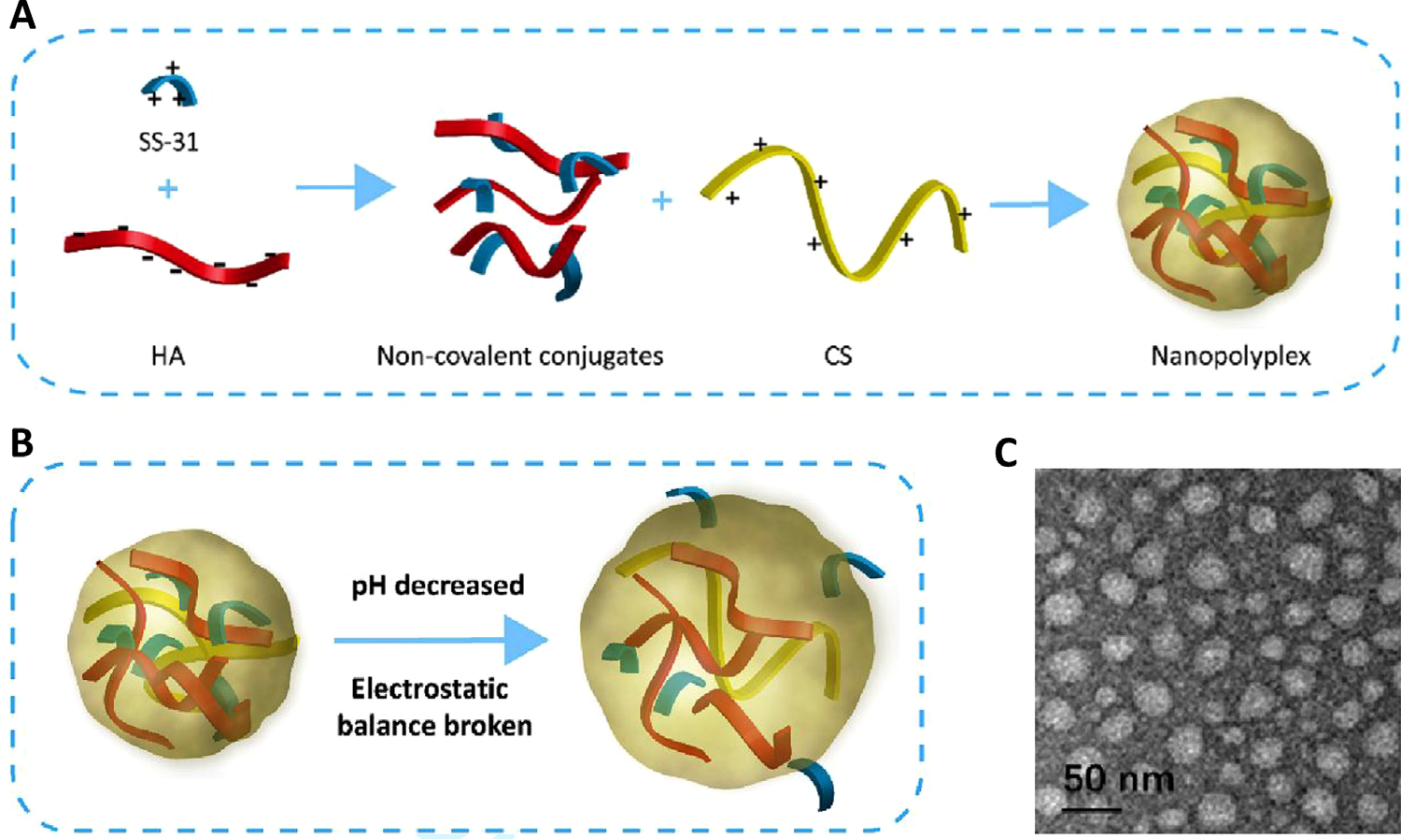
Engineered nanocarriers based on organic polymers: (A) Preparation methods for the nanocarrier based on hyaluronic acid and chitosan, loaded with SS‐31. (B) In a low pH environment, the nanocarrier would release its component, SS‐31. (C) Typical TEM images of the nanocarrier. Reproduced with permission.^[^ ^49^ ^]^ Copyright 2019, Elsevier.

SS‐31 is a mitochondria‐targeted peptide with intense antioxidant activity. However, due to the non‐specific biodistribution, frequent administration is necessary for effective treatment. Thus, researchers constructed a pH‐responsive nanocarrier targeting AKI‐kidney and CD44‐overexpressed cells (Figure 15). This nanocarrier is a formulation of hyaluronic acid chitosan, and it achieved satisfying levels of drug loading, encapsulation, and drug release efficiency. The size of the nanocarrier is approximately 53 nm, and thus, it cannot accumulate in normal kidneys. Furthermore, in vivo bioavailability experiment results demonstrated efficient oxidative stress alleviation capabilities and tubular apoptosis and necrosis prevention. While this nanocarrier has all the desirable attributes, its biotoxicity has not yet been rigorously examined. Since the particle could not penetrate normal GFB, it may result in over‐accumulation and cause significant adverse effects.^[^ ^49^ ^]^

In addition, Hong Sang Choi et al. loaded 1‐dodecanethiol stabilized Mn_3_O_4_ inside ROS‐sensitive nanomicelles (PTC) to prevent premature release during circulation and achieve ROS‐responsive release. The nanomicelles provide a convenient solution for selective in vivo release.^[^ ^65^ ^]^

#### 4.4.2 Nanocarriers based on inorganic materials

Apart from organic materials, inorganic materials are also popular in nanocarrier engineering. In addition to fundamental carrier properties, inorganic material‐based nanocarriers can exhibit a certain level of antioxidative ability. As described in the previous section, ceria nanoparticles exhibit strong and recyclable ROS scavenging activity and be broadly utilized to treat ROS‐related diseases. However, there are a couple of drawbacks: (i) ceria nanoparticles have a diameter of <5 nm, which is ideal for ensuring a high level of biomimetic enzyme activity but can also result in a short half‐life in blood circulation; and (ii) ceria nanoparticles cannot directly target mitochondria, which is a critical intracellular site for ROS production.^[^ ^77^ ^]^

**FIGURE 16:**
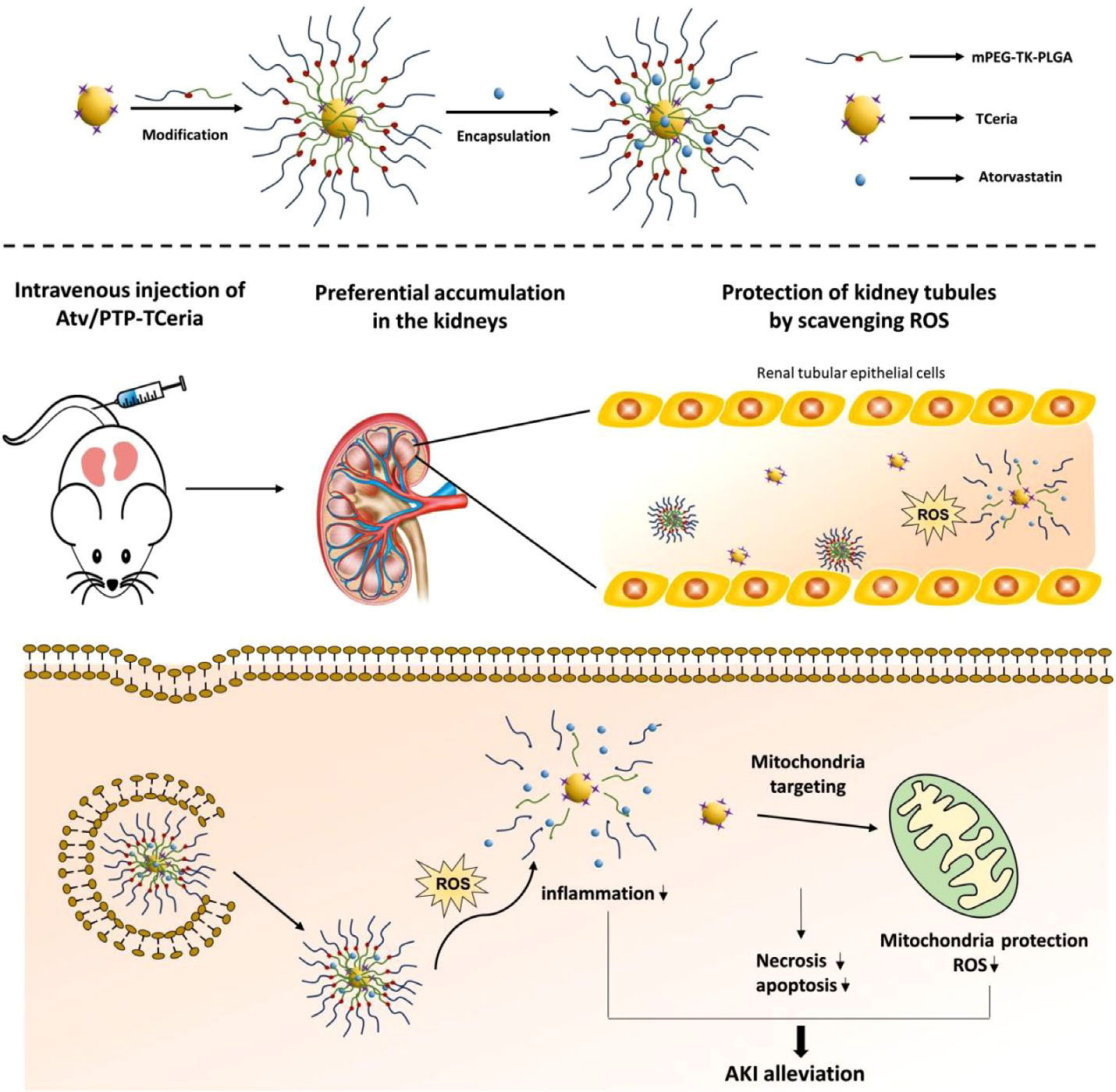
The nanocarrier can carry atorvastatin inside cells and reduce ROS inside mitochondria for protection. Additionally, the nanocarrier itself can eliminate in vivo ROS during the release of inner components. Reproduced under the terms of the CC‐BY license.^[^ ^78^ ^]^ Copyright 2020, Hui Yu et al.

To address these drawbacks and improve overall functionality, Hui Yu et al. have designed ROS‐responsive organic polymer coated ceria nanoparticles loaded with atorvastatin. In renal tubular areas, these nanoparticles can eliminate extracellular ROS and degrade into smaller particles, but after endocytosis, the nanoparticles were found to alleviate ROS stress inside the cells and mitochondria. (Figure 16) This desirable feature means that this nanodrug can significantly protect cells against ROS. The in vitro experiment demonstrated that combining these three components (ceria nanoparticles, organic polymers, and atorvastatin) has superior abilities to relieve ROS when compared to single or double component combinations.^[^ ^78^ ^]^ This research indicates that combining several materials with similar properties could result in surprising outcomes.

Additionally, while traditional ROS scavenging medicines have significant efficiency, their clinical applications are limited due to their poor specificities, low bioavailability, and high levels of bio toxicity. To address these issues, ultrasmall gold nanoclusters capped with traditional medicine (*N*‐acetylcysteine) have been developed. This constructed nanoparticle takes advantage of the intrinsic attributes of nanozymes, the physicochemical stability of gold, and the antioxidant ability of *N*‐acetylcysteine. Due to its small size, in vivo experiment results have shown multienzyme‐like activity and preferential renal uptake. Researchers have proposed a promising novel nanodrug for potential clinical use by analyzing the anti‐inflammatory and antioxidative abilities.^[^ ^79^ ^]^

## 5 CONCLUSIONS AND PROSPECTS

### 5.1 An ideal engineered nanodrug to target oxidative stress in AKI treatments

#### 5.1.1 Targeting

One of the significant challenges in traditional clinical medicine is unspecific biodistribution, especially of large nanoparticles. Thus, ideal nanodrugs should be able to target one specific area. Currently, most nanomedicines targeting the renal area also display high levels of accumulation in the liver and other areas. Ideally, a nanodrug should only display high accumulation levels in one area, thus minimizing the dosage and potential adverse effects. Based on current research, particles with small diameters (<10 nm) in at least one dimension are most likely to have specific accumulation in the kidney.

#### 5.1.2 Properties

Specific targeting ensures that nanoparticles reach the desired area, while their properties determine their efficacy in disease treatment. To achieve desired properties and maintain specific targeting, scientists have to enclose the functionalities inside tiny nanostructures, which is exceptionally challenging. A perfect nanomedicine should bear the desired property itself or load other medicines with the property and, in both cases, remain stable inside the harsh in vivo environment. Additionally, researchers must consider the differences between “biological identity” and “synthesis identity” to help ensure that the desired properties are intact upon reaching the target area.

#### 5.1.3 Biosafety

Biosafety is also a crucial concern when developing potential medicines. Most inorganic nanoparticles are developed based on heavy metals, which have a certain level of biotoxicity. Non‐biodegradable nanomaterials can accumulate in the liver and spleen for months to years, leading to toxicity concerns. Quick body excretion is seen as an acceptable solution to this issue.^[^ ^85^ ^]^ However, rapid biodegradation could result in low efficacy. Thus, balancing the scale between biosafety and efficacy is of utmost importance. Additionally, although metal‐based nanomaterials demonstrate desirable attributes, they are usually toxic.^[^ ^86^ ^]^ Furthermore, some nanomaterials are particularly reactive with serum proteins due to intense surface energy, leading to potential alterations in their biodistribution, excretion pathways, and biocompatibility. The biosafety experiments discussed in this article only provide rudimentary levels of data. Thus, thorough and detailed biosafety assessments will be required for any nanomedicine to proceed to further clinical trials.

#### 5.1.4 Mass preparation

Apart from being perfectly efficacious and safe, nanodrugs also need to be suitable for mass production. Ingeniously designed nanodrugs can be very challenging to synthesize. While some particles satisfy the first two criteria, they may also have complicated synthesis steps, which may fail even in a dedicated laboratory environment, resulting in unacceptable expenses. Thus, a simple, high‐productivity synthesis process is essential for large‐scale clinical usage. Although this property might not be significant in the early stages of drug development, it is why many modern medicines cannot be widely utilized.

#### 5.1.5 Clinical transformation

The ideal engineered nanodrugs targeting oxidative stress in AKI treatment should have excellent targeting capabilities, ROS scavenging capacity, good biosafety, and good mass production capability. At present, the reported engineered nanodrugs targeting oxidative stress in AKI therapy do not meet all the above conditions. For example, cerium dioxide nanoparticles with good ROS‐scavenging capacity can have excellent targeting capabilities after modification, which has potential application in the treatment of AKI. However, the ROS‐scavenging capacity of cerium dioxide nanoparticles is affected by the size, valency of Ce, specific surface area, and other factors; thus, it is still unable to achieve mass production. In addition, it cannot be degraded well in vivo and has potential long‐term toxicity. For bilirubin nanoparticles, they have good ROS scavenging performance and high biosafety. However, the problems of mass production and targeting need to be solved.

The ideal engineered nanodrugs targeting oxidative stress in AKI treatment should also adapt to other oxidative stress related diseases, such as liver injury (drug‐induced liver injury, fatty liver, liver ischemia reperfusion injury, etc.). However, the ultimate goal for engineering nanodrugs is similar: to create a nanomedicine with specific targeting, desired property, maximum biosafety, and capability for mass production.

### 5.2 Mechanism for engineering nanodrugs to target oxidative stress in AKI treatments

In the previous sections, we discussed the essential attributes of nanodrugs that can be utilized to treat AKI, including GFB and oxidative stress targeting. However, the exact mechanisms of the targeting capabilities remain unknown. Nanoscale particles have been confirmed to own unique qualities, yet the explanation for their distinctive in vivo behaviours remains debatable. To target the renal area, size has been recognized as a dominant factor to consider. However, it has been shown that particles of similar sizes can result in entirely different biodistributions, casting doubts on our initial beliefs. Additionally, a particular combination of antioxidants could enhance antioxidative capabilities, while other amalgamations may have different effects. The engineering process could be standardized if the basis for the in vivo attributes were elucidated, thus empowering researchers to construct nanoparticles with desired properties.^[^ ^87^ ^]^

### 5.3 Experimental models for AKI

In‐depth studies of diseases often utilize mature experimental models. However, most experimental models can only partially simulate human disease progress, which is especially relevant for AKI. Currently, the experimental models for AKI are limited to the kidney organoid, zebrafish, rodent, and large animal models. Unfortunately, the results of previous studies have shown that rodents cannot accurately represent the disease progression of human AKI, thus prohibiting basic research using this model from being transferred into clinical practice. Thus, an accurate model representing the specific disease would greatly benefit AKI nanodrug development. Nevertheless, though the current experimental models have limitations, scientists must still rely on them for fundamental research for the foreseeable future. Consequently, a robust theory and thorough clinical trial are essential before clinical applications.

This review has summarized GFB‐targeting methods and analysed recent attainments in the development of AKI nanodrugs. However, numerous additional investigations will be inevitable to put this research into practice. To aid in this process, this review has also outlined the current achievements and drawbacks in this field of research to guide future research and experimental design.

## CONFLICT OF INTEREST STATEMENT

The authors declare no conflicts of interest.
